# Inhibition of Cathepsin S Reduces Lacrimal Gland Inflammation and Increases Tear Flow in a Mouse Model of Sjögren’s Syndrome

**DOI:** 10.1038/s41598-019-45966-7

**Published:** 2019-07-02

**Authors:** Wannita Klinngam, Srikanth R. Janga, Changrim Lee, Yaping Ju, Frances Yarber, Mihir Shah, Hao Guo, Dandan Wang, J. Andrew MacKay, Maria C. Edman, Sarah F. Hamm-Alvarez

**Affiliations:** 1 Department of Pharmacology and Pharmaceutical Sciences, School of Pharmacy, University of Southern California, Los Angeles, CA 90033 USA; 2 Department of Ophthalmology, Roski Eye Institute, Keck School of Medicine, University of Southern California, Los Angeles, CA 90033 USA; 3 Anatomic and Clinical Pathology, Los Angeles County + University of Southern California Medical Center, Los Angeles, CA 90033 USA; 4 Department of Biomedical Engineering, Viterbi School of Engineering, University of Southern California, Los Angeles, CA 90089 USA

**Keywords:** Autoimmune diseases, Lacrimal apparatus diseases

## Abstract

Cathepsin S (CTSS) is highly increased in Sjögren’s syndrome (SS) patients tears and in tears and lacrimal glands (LG) of male non-obese diabetic (NOD) mice, a murine model of SS. To explore CTSS’s utility as a therapeutic target for mitigating ocular manifestations of SS in sites where CTSS is increased in disease, the tears and the LG (systemically), the peptide-based inhibitor, Z-FL-COCHO (Z-FL), was administered to 14–15 week male NOD mice. Systemic intraperitoneal (i.p.) injection for 2 weeks significantly reduced CTSS activity in tears, LG and spleen, significantly reduced total lymphocytic infiltration into LG, reduced CD3+ and CD68+ cell abundance within lymphocytic infiltrates, and significantly increased stimulated tear secretion. Topical administration of Z-FL to a different cohort of 14–15 week male NOD mice for 6 weeks significantly reduced only tear CTSS while not affecting LG and spleen CTSS and attenuated the disease-progression related reduction of basal tear secretion, while not significantly impacting lymphocytic infiltration of the LG. These findings suggest that CTSS inhibitors administered either topically or systemically can mitigate aspects of the ocular manifestations of SS.

## Introduction

Sjögren’s syndrome (SS) is a systemic autoimmune disease associated with inflammation of lacrimal gland (LG) and salivary gland (SG), resulting in severe dryness of the eyes and mouth^1^. The clinical presentation of SS is heterogenous and varies from sicca symptoms and LG and SG lymphocytic infiltration, to B-cell lymphoma and severe systemic involvement associated with lung disease, renal failure, cryoglobulinaemic vasculitis, and cardiovascular disease^2^. The non-obese diabetic (NOD) mouse is a well-established and extensively studied animal model of SS which spontaneously develops LG and SG lymphocytic infiltration associated with reduced secretory flow^3,4^. The male NOD mouse exhibits severe LG inflammation (dacryoadenitis) by 12–20 weeks and is commonly used as a model for studying ocular manifestations of SS, while the female NOD mouse develops dacryoadenitis after 30 weeks^4,5^. Our previous studies showed that cathepsin S (CTSS), a lysosomal cysteine endopeptidase involved in immune responses, is highly increased in tears and inflamed LG of 12-week-old male NOD mice relative to age matched male BALB/c mice^6^. In addition, CTSS activity is significantly elevated in SS patients tears compared with levels in tears of healthy control subjects or patients with non-SS-related dry eye or non-SS autoimmune diseases, suggesting elevated tear CTSS may uniquely distinguish SS patients^7,8^.

CTSS plays an important role in class II major histocompatibility complex (MHC) – mediated immune responses, specifically the degradation of the invariant chain (Ii), an MHC II associated protein that blocks antigenic peptide binding^9,10^. CTSS cleaves Ii, generating class II-associated invariant chain peptides (CLIP) which are replaced by HLA-DM (in human) or H-2M (in mice), allowing MHC II to represent antigen to CD4+ T-cells^11^. CTSS-deficient mice exhibit defective invariant chain (Ii) processing in dendritic cells and B cells, including the impairment of antigenic peptide presentation, suggesting that CTSS is essential for antigen presentation and further that inhibition of CTSS may be useful for treatment autoimmune diseases such as rheumatoid arthritis, systemic lupus erythematosus (SLE), and multiple sclerosis^12–14^.

CTSS also cleaves protease-activated receptor-2 (PAR-2) at a distinct N-terminal site generating a novel tethered ligand that can induce neurogenic inflammation and pain^15,16^, glomerular endothelial injury and microvascular permeability^13,17^, atopic dermatitis^18^, and activation of liver tumour-initiating cells associated with hepatocellular carcinoma^19^. CTSS at activity levels comparable to that in tears of SS patients increases gene and protein expression of PAR-2 in human corneal epithelial cells (HCE T-cells), while inducing secretion of IL-6, TNF-α, IL-1β, and MMP-9 in pathways mediated at least in part by PAR-2^20^. These findings suggest that tear CTSS, as well as LG CTSS, may both represent potential therapeutic targets for amelioration of LG and ocular surface inflammation in SS patients.

Here, this study tested whether CTSS activity contributes to autoimmune dacryoadenitis and reduced tear production using the male NOD mice as a model of the ocular symptoms of SS. We hypothesised that if elevated CTSS is involved in disease progression in either tear production and/or LG inflammation, then the inhibition of its activity would improve tear flow and/or reduce autoimmune dacryoadenitis. In this study, the CTSS inhibitor, Z-FL-COCHO (Z-FL), was administered i.p. and topically as an eyedrop. Z-FL is a synthetic peptide-based CTSS inhibitor composed of phenylalanine and leucine^21^. It exhibits slow binding and reversible kinetics with 400-fold more selectivity for CTSS (Ki = 0.185 nM) than for cathepsin B (Ki = 76 nM)^21^. Z-FL has been administered intraperitoneally (i.p.) in a mouse model for treatment of CD4+ T-cell-associated neuropathic pain and in an experimental mouse model of liver injury for treatment of natural killer T cell-associated hepatitis^22,23^.

Our intent was to investigate whether CTSS inhibition could modulate symptoms of SS-like autoimmune dacryoadenitis using systemic or topical Z-FL as proof-of-principal, not to determine which administration modality was preferable, nor to determine the ideal dosage regimen, both of which would require pharmacokinetics evaluation. Both administration modalities were chosen since CTSS may have systemic (e.g., LG) and local (ocular surface) effects in autoimmune dacryoadenitis, and only by utilising topical administration could we guarantee that Z-FL reached the pool of CTSS existing in tears. In this study, we provide the first evidence that CTSS inhibition can mitigate many symptoms of autoimmune dacryoadenitis. Administration of Z-FL i.p. every other day for 2 weeks into 14–15 week diseased male NOD mice reduced CTSS enzymatic activity in tears, LG, and spleen while decreasing total lymphocytic infiltration and CD3+ and CD68+ cell infiltration of the LG while increasing stimulated tear secretion. When given topically as an eyedrop for 6 weeks, twice daily, in 14–15 week male NOD mice, tear CTSS activity and the reduced tear secretion that occurs with disease progression over this treatment regimen were simultaneously attenuated. These findings implicate CTSS as an effector of autoimmune dacryoadenitis, representing a viable therapeutic target for systemic and local administration in treatment of SS-associated dry eye.

## Results

### Systemic administration of Z-FL given intraperitoneally (i.p.)

#### Identification of i.p. doses of Z-FL

To estimate the Maximum Tolerated Dose (MTD) for Z-FL administered i.p., a dose-escalation study was conducted with Z-FL dissolved in vehicle (10% DMSO + 40% PEG 300 + 50% sterile PBS) at 0.25, 0.5, 1.0, 2.0 and 4.0 mg/kg body weight (BW). This vehicle was chosen from several formulations as non-toxic while best able to dissolve this hydrophobic agent; 4.0 mg/kg was the highest dose dissolved. Z-FL concentrations at each dose were 140 µM, 290 µM, 580 µM, 1.1 mM and 2.3 mM, respectively, and determined using reverse-phase high performance liquid chromatography (RP-HPLC). Male BALB/c mice were injected with volumes of 100–120 µl, depending upon mouse BW, every other day for 2 weeks. During this protocol, no weight loss >15% of starting BW was observed. After 2 weeks, no notable difference in BW nor in the weight or appearance of spleen, liver or kidney in any treatment groups was observed **(**Supplementary Fig. S1**)**. As no signs of toxicity were observed at any dose, for the purpose of this study, the highest evaluated dose of 4 mg/kg was considered as the MTD, while 1 mg/kg was evaluated as an intermediate dose for a therapeutic i.p. injection protocol.

#### Intraperitoneal Z-FL reduces CTSS activity in tears, LG and spleen lysates in male NOD mice

Z-FL was administered i.p., every other day for 2 weeks, to 14–15 week male NOD mice, at which time autoimmune dacryoadenitis is typically established^6^. As NOD male mice can develop type 1 diabetes with a usual onset between 16–24 weeks of age^24^, blood glucose was measured before and after treatment. Mice with blood glucose levels >250 mg/dl were considered to have developed diabetes during the study and were excluded^25–27^. No mice in the i.p. cohort had blood glucose levels >250 mg/dl at the conclusion of the study, so the number of mice in each of these cohorts remained 10.

**Figure 1:**
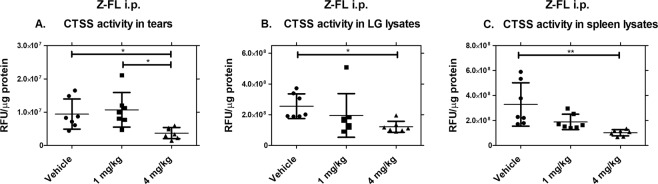
Intraperitoneal Z-FL reduces CTSS activity in tears, LG lysates, and spleen lysates. 14–15 week old male NOD mice were treated every other day for 2 weeks with i.p. Z-FL at 1, 4 mg/kg body weight. **(A)** Tears were assayed for CTSS activity, which showed 4 mg/kg Z-FL significantly reduced activity relative to 1 mg/kg and vehicle (*P* = 0.04 and *P* = 0.02 respectively); **(B)** LG lysates were assayed for CTSS activity, which showed that only Z-FL at 4 mg/kg reduced activity relative to vehicle (*P* = 0.046); **(C)** Spleen lysates were assayed for activity, and only 4 mg/kg reduced activity relative to vehicle (*P* = 0.003). N = 7 mice/group, data are represented as mean ± SD; a one-way ANOVA with Tukey’s multiple comparison was used to compare treatments.

To investigate how systemic Z-FL administration given i.p. affected CTSS activity, we measured CTSS activity in tears, LG and spleen. CTSS activity in tears from mice treated with 4 mg/kg of Z-FL i.p. was reduced significantly relative to mice treated with 1 mg/kg of Z-FL or vehicle **(**Fig. 1A**)**. For CTSS activity in LG lysates, 4 mg/kg Z-FL treated mice showed less CTSS activity relative to CTSS activity in the vehicle-treated group **(**Fig. 1B**)**. Additionally, 4 mg/kg Z-FL also significantly reduced CTSS activity in spleen lysates relative to levels in vehicle group **(**Fig. 1C). 1 mg/kg Z-FL did not reduce CTSS activity in tears, LG or spleen. **(**Fig. 1A–C**)**. These findings suggest that i.p. Z-FL at 4 mg/kg affects both local (ocular surface) and systemic (LG, spleen) CTSS activity levels.

#### Intraperitoneal Z-FL decreases LG lymphocytic infiltration and increases stimulated tear secretion in male NOD mice

**Figure 2:**
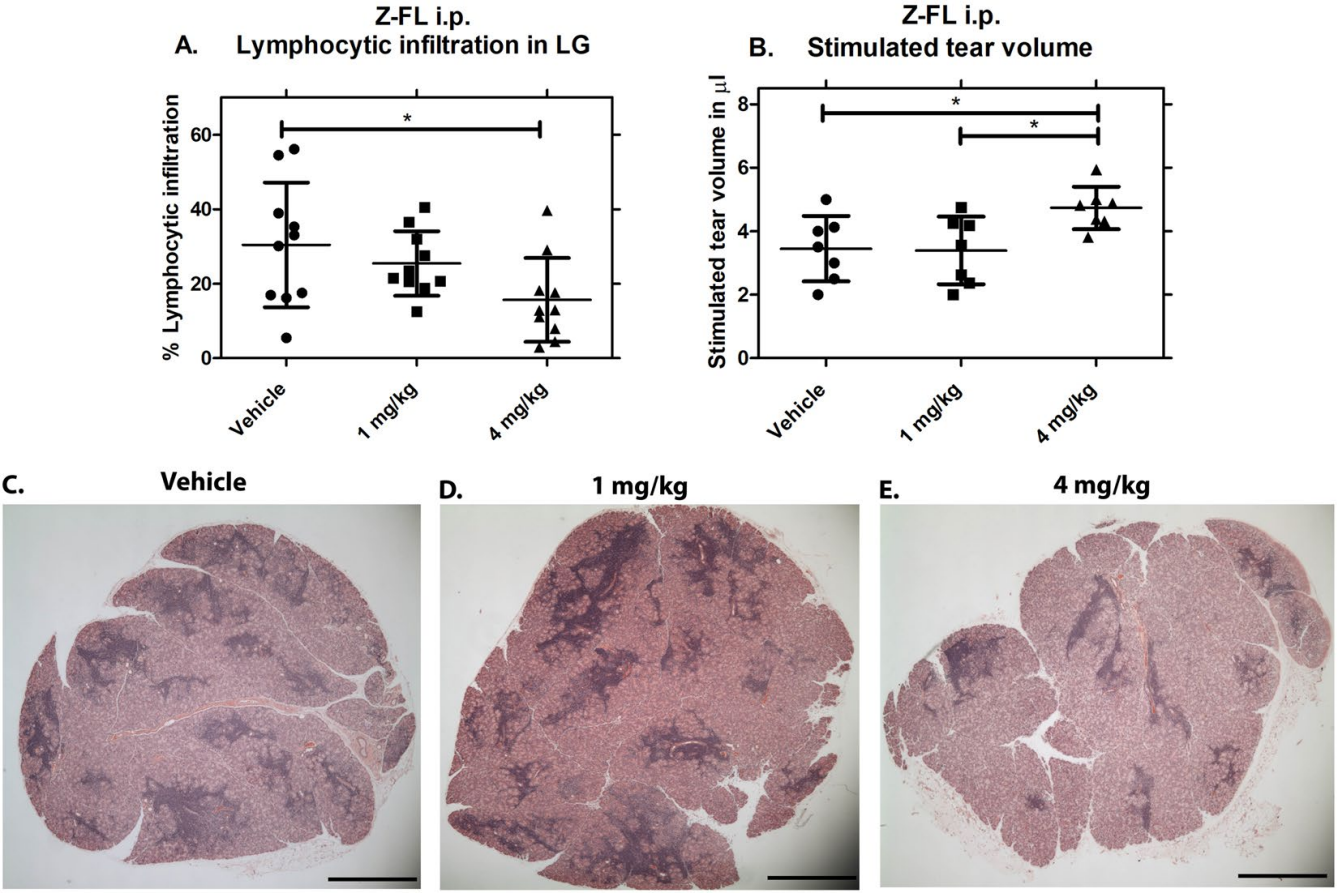
Intraperitoneal Z-FL decreases lymphocytic infiltration of the LG and increases stimulated tear secretion. 14–15 week old male NOD mice were treated every other day for 2 weeks with i.p. Z-FL at 1, 4 mg/kg body weight. **(A)** The percentage of LG area occupied by lymphocytic infiltration was significantly reduced by 4 mg/kg Z-FL compared to vehicle alone (n = 10 mice/group, *P* = 0.04); **(B)** Carbachol-stimulated tear secretion was assessed, and 4 mg/kg of Z-FL significantly increased tear production relative to vehicle or 1 mg/kg treatment groups (n = 7 mice/group, *P* = 0.049, *P* = 0.04 respectively). Data are represented as mean ± SD, a one-way ANOVA with Tukey’s multiple comparison was used to compare between treatment groups; **(C**–**E)** Representative hematoxylin/eosin stained section of LG are provided from mice treated with **(C)** vehicle; **(D)** 1 mg/kg Z-FL; **(E)** 4 mg/kg Z-FL. Scale bar = 400 µm.

The percentage of LG area infiltrated by lymphocytes was determined after each treatment. 4 mg/kg of Z-FL given i.p. significantly reduced LG inflammation relative to vehicle, while 1 mg/kg did not have an effect **(**Fig. 2A). Figure 2C–E shows representative images of LG cross sections from mice exposed to i.p. Z-FL and vehicle. Additionally, to investigate whether Z-FL administered systemically could improve tear secretion, stimulated tear secretion was measured at study conclusion by adding topical carbachol to the exposed LG. 4 mg/kg of Z-FL i.p. increased stimulated tear secretion relative to 1 mg/kg Z-FL and vehicle, while 1 mg/kg Z-FL had no effect **(**Fig. 2B**)**.

#### Intraperitoneal Z-FL reduces CD3+ and CD68+ cell abundance in lymphocytic infiltrates in parallel with reduced MHC II (*H2-Ab1)* gene expression in LG

**Figure 3:**
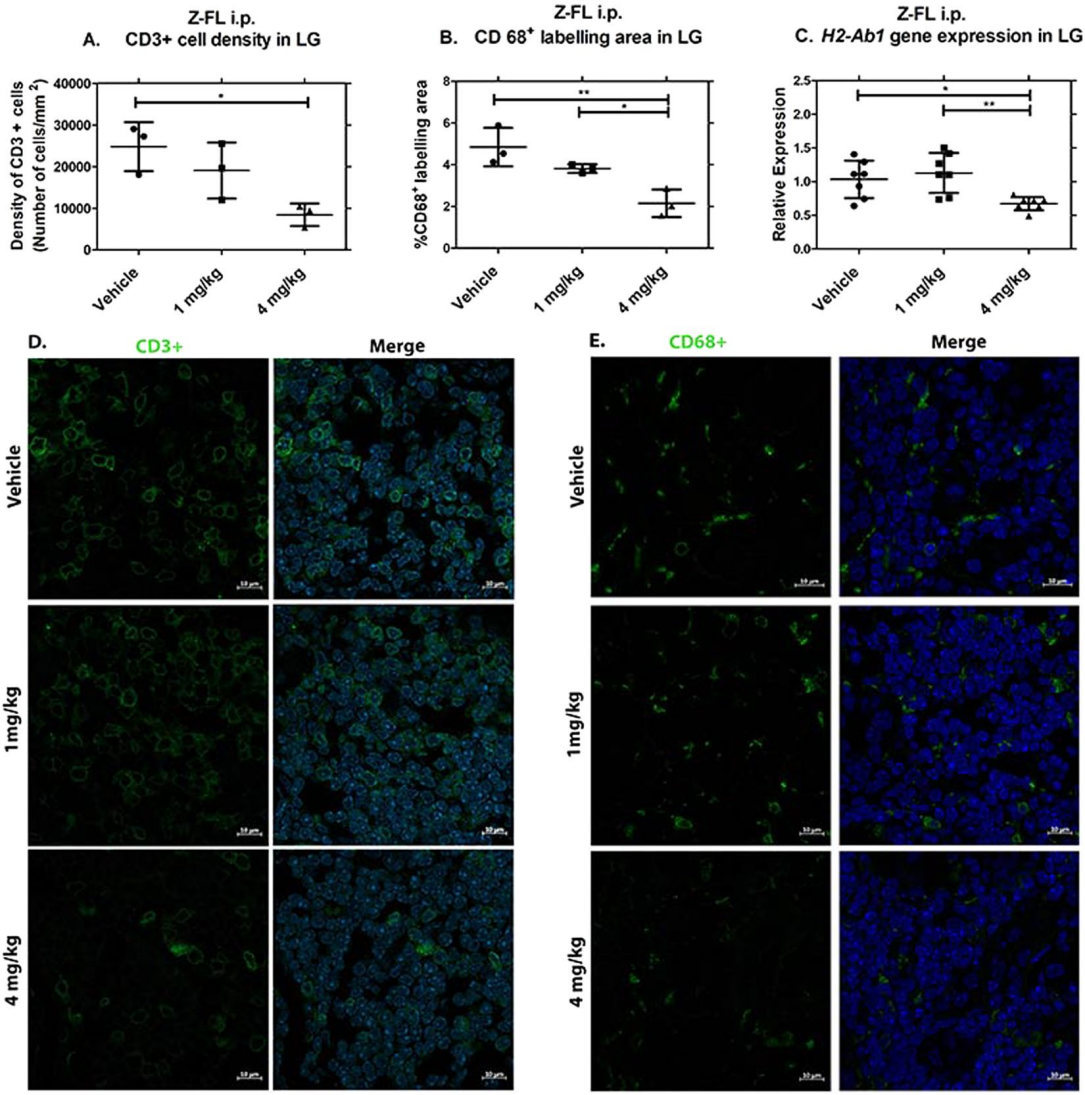
Intraperitoneal Z-FL reduces CD3+ cell and CD68+ cell abundance in lymphocytic infiltrates in parallel with reduced MHC II (*H2-Ab1)* gene expression in LG. 14–15 week old male NOD mice were treated every other day for 2 weeks with i.p. Z-FL at 1, 4 mg/kg body weight. (**A**) LG were assessed for density of CD3+ cells in areas of lymphocytic infiltration, and the group treated with 4 mg/kg Z-FL had significantly lower than vehicle alone (n = 3 mice/group*, P* = 0.02); (**B**) LG were assessed for the percentage of CD68+ cells in areas of lymphocytic infiltration, and the group treated with 4 mg/kg Z-FL had significantly fewer CD68+ cells compared to vehicle alone or 1 mg/kg of Z-FL (n = 3 mice/group, *P* = 0.006, *P* = 0.05 respectively); (**C**) LG homogenates were assessed for *H2-Ab1* gene expression normalised to endogenous *Gapdh*, and only 4 mg/kg Z-FL significantly reduced expression relative to vehicle or 1 mg/kg of Z-FL (n = 7 mice/group, *P* = 0.03, *P* = 0.007 respectively). Data represent mean ± SD, a one-way ANOVA with Tukey’s multiple comparison was used to compare between mouse groups. (**D**) Representative images of CD3+ cell immunostaining in areas of lymphocytic infiltration from treatment groups in (**A**). CD3 (Green) labels total T-cells while DAPI labels nuclei (blue); (**E**) Representative LG images of CD68+ cell immunostaining in areas of lymphocytic infiltration from treatment groups in (**B**). CD68 (Green) labels pan-macrophages, dendritic cells and progenitor monocytes and DAPI (Blue) labels nuclei. Scale bar = 10 µm.

Our previous work has shown that the immune cell infiltrates in the LG of male NOD mice at 18 weeks include T-cells, macrophages, B-cells, and smaller populations of other cells^28^. We evaluated how Z-FL, administered i.p., affected the major immune cell populations. Utilising CD3 as a marker for T cells at all stages of development^29^, the density of CD3+ cells (number of cells/total area of cells) in areas of lymphocytic infiltration was measured under each condition. In LG from mice given 4 mg/kg of Z-FL i.p., CD3+ cell density was significantly reduced relative to LG from vehicle-treated mice (Fig. 3A, representative images in Fig. 3D).

To investigate whether i.p. Z-FL affected the number of CTSS-enriched antigen-presenting cells such as macrophages and dendritic cells (DC) in the lymphocytic infiltrates, CD68 was used as a marker for pan-macrophages, inflammatory macrophages (M1), anti-inflammatory macrophages (M2), DC and monocytes which differentiate into these cell types^30–36^. As shown in Fig. 3B (representative images in Fig. 3E), 4 mg/kg of i.p. Z-FL decreased CD68+ cells in lymphocytic infiltrates, reported as the percentage of CD68+ labelling area and normalised to the total area of lymphocytic infiltrates in each image field, relative to 1 mg/kg and vehicle. Comparable analysis of the effects of i.p. Z-FL using an antibody to B220, a pan-B-cell marker^37^, showed no differences in B220+ cells in lymphocytic infiltrates among treatment groups **(**Supplementary Fig. S2**)**.

CTSS cleaves Ii, required for MHC II molecules to bind antigenic peptides^10^. MHC II is typically expressed in professional antigen presenting cells such as macrophages and DC, as well as B lymphocytes^38^. *H2-Ab1* gene expression in LG of 4 mg/kg Z-FL treated mice was significantly reduced relative to LG treated with 1 mg/kg Z-FL and vehicle **(**Fig. 3C**)**. This reduction paralleled the reduction in CD68+ cell content within the LG seen with i.p. Z-FL.

#### Intraperitoneal Z-FL does not affect expression of other inflammation-associated genes in LG of male NOD mice

Our previous work found that CTSS, TNF-α, and IFN-γ were significantly increased in NOD mouse LG during development of autoimmune dacryoadenitis^6,39^. CTSS also increases TNF-α and PAR-2 gene and protein expression in cultured human corneal epithelial cells, suggesting that its activity may drive ocular surface inflammation^20^. We analysed whether these additional CTSS-associated genes were affected in LG of mice treated with i.p. Z-FL. Beyond *H2-Ab1*, expression of other genes linked to CTSS activity including *Ctss* itself, *Tnf*, *Ifng*, and *F2rl1* were unchanged by i.p. Z-FL at either dose (Supplementary Fig. S3).

#### Intraperitoneal Z-FL does not elicit gross systemic toxicity at the dose evaluated

The spleen, liver, and kidneys of treated mice were evaluated for tissue toxicity by a trained pathologist following all treatments. The data showed that there was not any statistical association between kidney or liver findings vs mouse treatment groups. The mild diffuse vacuolisation of the tubular epithelial cells that was found in mice exposed to 1 mg/kg and 4 mg/kg of Z-FL given i.p. is normally present in the tubules of male mice^40^. There was no notable difference in the number and/or size of vacuoles compared to vehicle-treated mice suggesting that drug does not elicit kidney abnormalities. Also, the focal cytoplasmic swelling and vacuolisation noted in one vehicle-treated mouse, two mice treated with 1 mg/kg Z-FL i.p., and three mice treated with 4 mg/kg Z-FL i.p. reflect nonspecific changes in liver cells reflective of factors such as ischemia, or changes in the diet or metabolic condition of the mice^41^ **(**Supplementary Table S1**)**.

### Topical administration of Z-FL

#### Identification of topical doses of Z-FL

**Figure 4:**
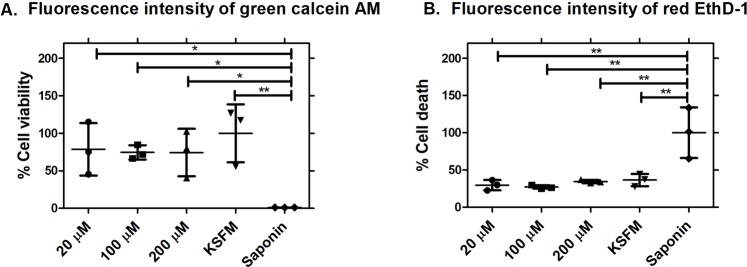
Z-FL does not reduce cell viability or cause cell death *in vitro* in human corneal epithelial (HCE-T) cells. (**A**) The percentage of cell viability at all doses tested of Z-FL is plotted relative to that seen with KSFM treatment (cultured medium) and saponin treatment. *P* = 0.04 (20 µM vs saponin), *P* = 0.02 (100 µM vs saponin), *P* = 0.046 (200 µM vs saponin), and *P* = 0.007 (KSFM vs saponin); **(B)** The percentage of cell death at all doses of Z-FL tested is plotted relative to saponin treatment, and KSFM treatment (cultured medium). *P* = 0.002 (20 µM vs saponin), *P* = 0.002 (100 µM vs saponin), *P* = 0.004 (200 µM vs saponin), and *P* = 0.005 (KSFM vs saponin). Cells treated with KSFM were positive controls for live cells, while cells treated with saponin were positive controls for dead cells. N = 3 wells/group, data shown as mean ± SD, and a one-way ANOVA with Tukey’s multiple comparison was used to compare between treatment groups).

To provide an initial estimate of the dose of topical Z-FL that would not elicit corneal epithelial cell toxicity, cell viability and cytotoxicity were assessed in the human corneal epithelial cell line transformed with Simian virus 40-adeno vector (HCE-T cells^42^) using 20, 100, and 200 µM of Z-FL in Keratinocyte-SFM (KSFM) medium. Cells treated with KSFM were positive controls for live cells, while cells treated with saponin were positive controls for dead cells. Z-FL treatment showed no differences at any dose in cell viability, measured as using green Calcein AM fluorescence intensity (Fig. 4A) relative to KSFM-treated cells. Conversely, saponin treatment increased cell death (measured as red Ethidium Homodimer-1 or EthD-1 fluorescence intensity) distinct from KSFM-treated cells and Z-FL treatment (Fig. 4B**)**. There was a significant difference between Z-FL-treated cells at all doses relative to saponin-treated cells, but no significant difference between KSFM-treated cells and all doses of Z-FL treatment, suggesting that Z-FL did not cause cell death.

Additionally, to estimate the optimal dose of Z-FL eye drops that were not associated with gross toxicity, we conducted a 1-week pilot eye drop study in 14-week male NOD mice. These same doses of 20, 100, and 200 µM Z-FL (200 µM being the highest concentration of Z-FL soluble in PBS) were administered to mouse eyes twice daily in sterile PBS for 1 week. None of these doses of Z-FL elicited punctate epithelial erosions on the ocular surface nor caused excessive tearing. No discharge was observed. PBS alone also did not elicit any of these symptoms. Other more traditional toxicology studies (including clinical scores at defined periods of time) were not performed at this time, but can be evaluated in future studies. Basal tear secretion measurements and corneal fluorescein staining results verified the lack of change between treatment groups at any dose of Z-FL relative to vehicle (PBS) **(**Supplementary Fig. S4A,B**)**. Thus, 200 µM of Z-FL, the highest dose in this pilot study, was chosen for topical administration during subsequent therapy studies.

#### Topical instillation of Z-FL reduces CTSS activity in tears of male NOD mice

**Figure 5:**
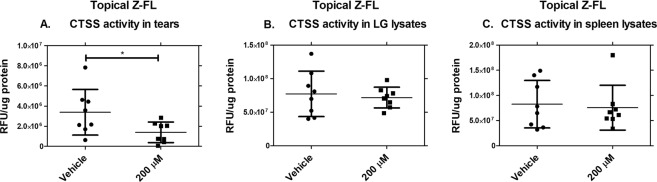
Topical instillation of Z-FL reduces CTSS activity locally in tears, but not in LG or spleen lysates. 14–15 week old male NOD mice were treated twice a day for 6 weeks with 200 µM Z-FL. **(A)** Tear samples were obtained, CTSS activity was assessed, and Z-FL-treatment significantly decreased activity relative to the vehicle alone (*P* = 0.04); **(B)** LG lysates were prepared, CTSS activity was assessed, and no differences were observed upon Z-FL-treatment; **(C)** Spleen lysates were prepared, CTSS activity was assessed, and no differences were observed upon Z-FL treatment. N = 8 mice/group, data are represented as mean ± SD, and a two-tailed, unpaired Student’s *t*-test was used to compare between mouse groups.

Z-FL was administered topically, twice daily for 6 weeks, to 14–15 week male NOD mice. Blood glucose was measured before and after treatments to exclude diabetic mice with blood glucose levels >250 mg/dl, which might complicate interpretation of the results^25–27^. 3 mice given topical eye drops (2 from the PBS group and 1 from the Z-FL group) were excluded at the conclusion of treatments, generating cohorts of 14 mice for 200 µM Z-FL group and 13 mice for the vehicle group. Tear, LG, and spleen CTSS activity were measured after 6-weeks of treatment with results showing reduction of tear CTSS activity by 200 µM of topical Z-FL compared to vehicle-treated mice **(**Fig. 5A**)**. Topical Z-FL did not inhibit systemic CTSS as reflected by lack of inhibition of LG (Fig. 5B) or spleen CTSS **(**Fig. 5C**)**.

#### Topical instillation of Z-FL does not affect total lymphocytic infiltration in LG of male NOD mice nor improve stimulated tear secretion but reduces CD68+ cell abundance in parallel with MHC II (*H2-Ab1)* gene expression

**Figure 6:**
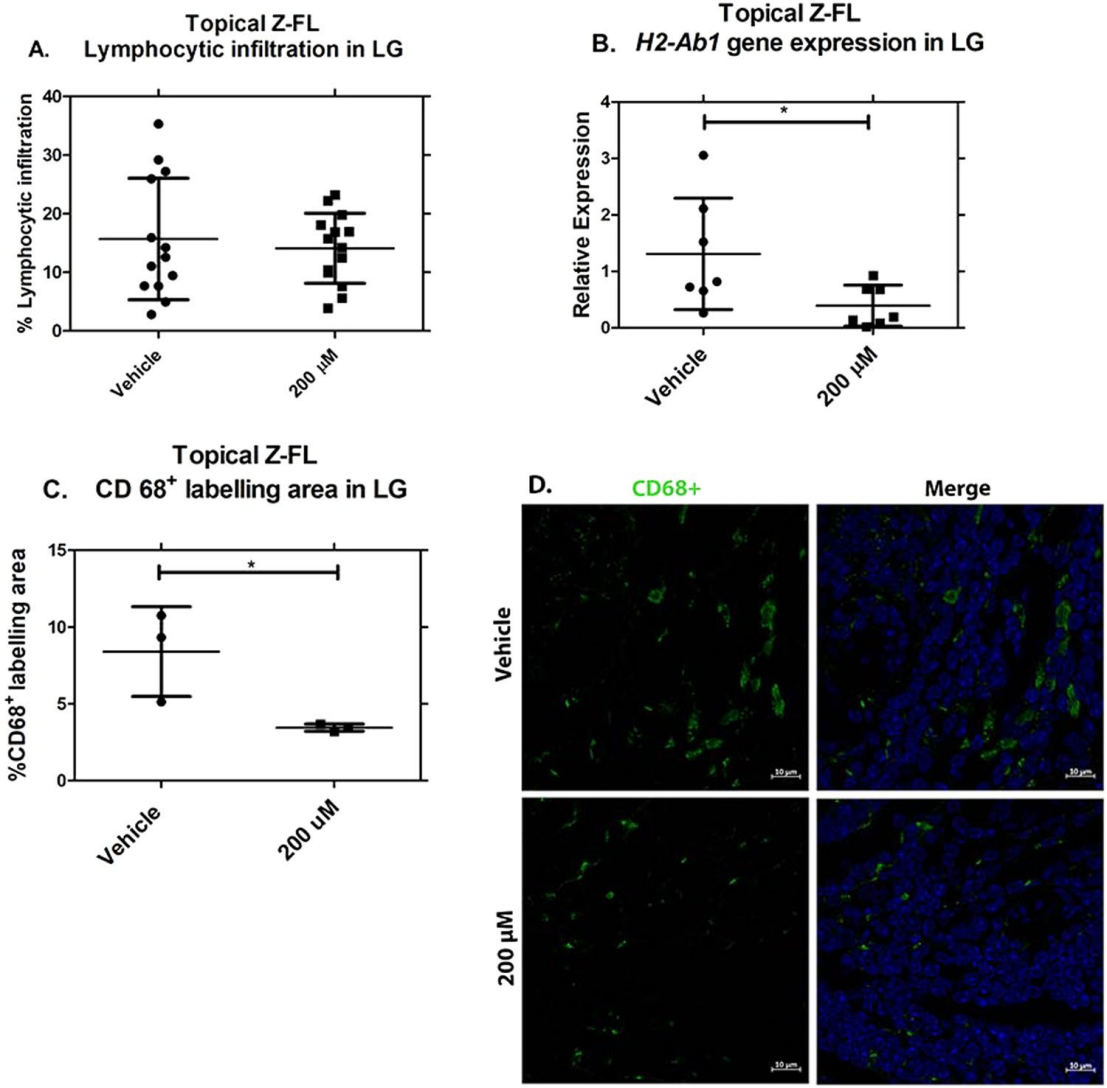
Topical instillation of Z-FL reduces CD68+ cell abundance and expression of MHC II (*H2-Ab1)* in LG, but does not affect LG lymphocytic infiltration. 14–15 week old male NOD mice were treated twice a day for 6 weeks with 200 µM Z-FL. (**A**) LG were obtained and assessed for area occupied by lymphocytic infiltration. Topical treatment with Z-FL was no different from vehicle. (n = 13, 14 for vehicle, drug groups respectively); (**B**) LG lysates were prepared and assessed for *H2-Ab1* gene expression. Treatment with Z-FL significantly reduced gene expression relative to vehicle (n = 7/group, *P* = 0.04). Expression of *H2-Ab1* was normalised to endogenous *Gapdh*; (**C**) LG were obtained, sectioned, assessed by immunofluorescence for CD68, and quantified. The percentage of CD68+ cells in areas of lymphocytic infiltration were significantly lower upon treatment with Z-FL compared to vehicle alone (n = 3/groups, *P* = 0.04); (**D**) Representative LG images of CD68+ cell immunostaining in areas of lymphocytic infiltration quantified in (**C**). CD68 (Green) labels pan-macrophages, dendritic cells and progenitor monocytes and DAPI (Blue) labels nuclei. Scale bar = 10 µm. Data represent mean ± SD. A two-tailed, unpaired Student’s *t*-test was used to compare treatments.

To examine whether topical delivery of Z-FL could reduce LG inflammation, the percentage of LG area infiltrated by lymphocytes was determined. 200 µM of topical Z-FL did not affect LG inflammation relative to vehicle **(**Fig. 6A**)**. The percentage of LG lymphocytic infiltration of 20-week old mice in this topical study was comparable to that reported in other previous studies using mice of the same age^43–45^. However, gene expression data revealed a 70% reduction of *H2-Ab1* gene expression in LG from topical Z-FL-treated mice compared to vehicle (Fig. 6B). Therefore, the abundance of immune cell populations in LG infiltrates was measured utilising immunofluorescence as in Fig. 3. 200 µM of topical Z-FL reduced CD68+ cell infiltrates relative to vehicle **(**Fig. 6C, representative images in Fig. 6D**)**. In contrast, no differences in CD3+ cells nor B220+ cells in lymphocytic infiltrates among any mouse treatment groups were seen **(**Supplementary Figs S5–6). No increase in stimulated tear secretion in Z-FL treated mice relative to vehicle-treated mice was noted **(**Supplementary Fig. 7**)**.

#### Topical instillation of Z-FL significantly reduces *Ctss*, *Tnf*, *Ifng*, and *F2rl1* gene expression in male NOD mouse LG

**Figure 7:**
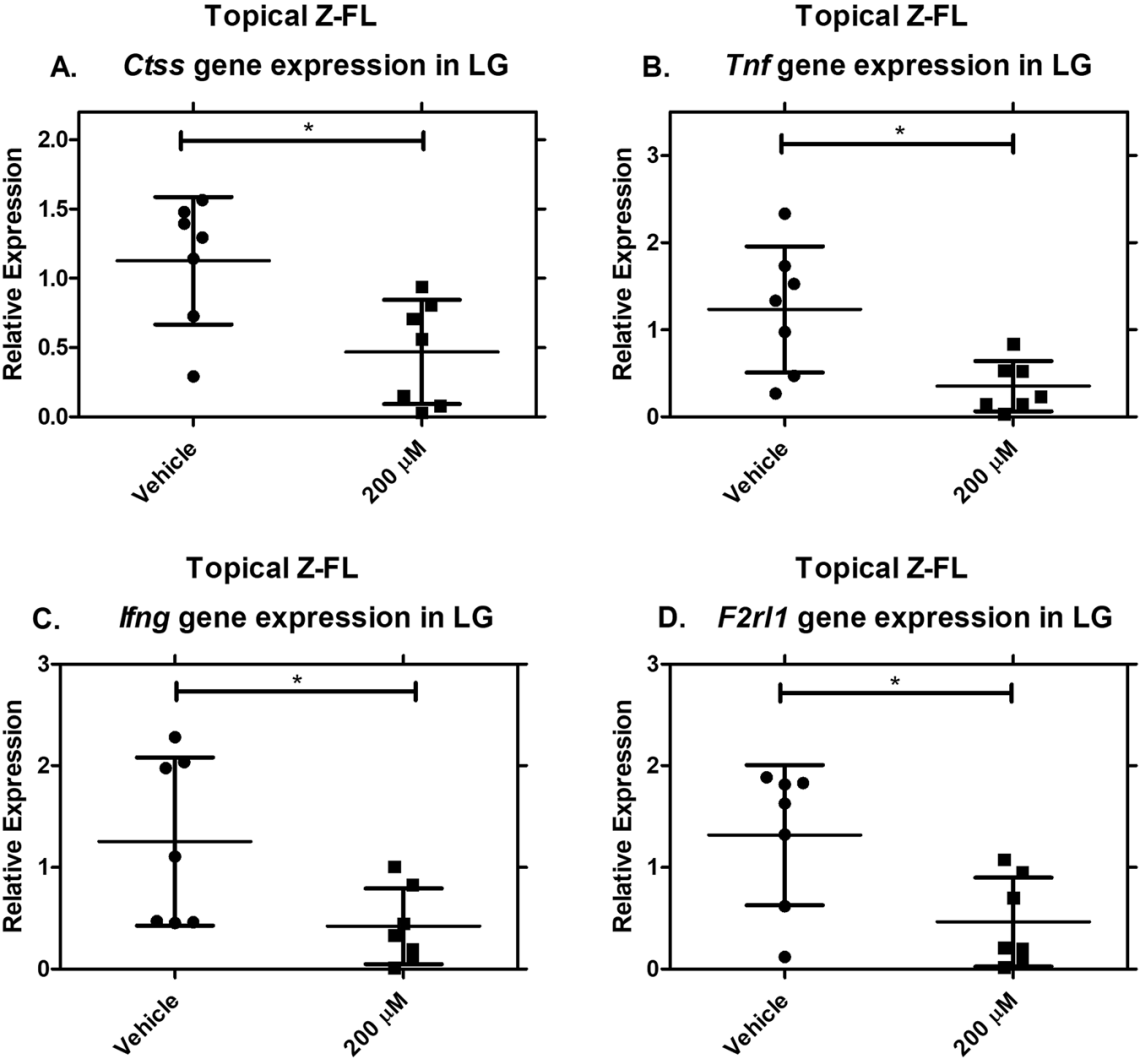
Topical instillation of Z-FL significantly reduces gene expression of *Ctss*, *Tnf*, *Ifng*, and *F2rl1*. 14–15 week old male NOD mice were treated twice a day for 6 weeks with 200 µM Z-FL. LG lysates were obtained and assayed for gene expression levels of **(A)** *Ctss* (*P* = 0.01); **(B)** *Tnf* (*P* = 0.01); **(C)** *Ifng* (*P* = 0.03); **(D)** *F2rl1* (*P* = 0.02). Expression of genes of interest was normalised to expression of the endogenous gene, *Gapdh*. N = 7 mice/group, data represent mean ± SD. A two-tailed, unpaired Student’s *t*-test was used to compare between mouse groups.

We analysed whether other CTSS-associated inflammatory genes were affected in LG in mice treated with topical Z-FL. Exposure of male NOD mice to Z-FL topical eye drops for 6 weeks significantly decreases LG gene expression of *Ctss*, *Tnf*, *Ifng*, and *F2rl1* relative to mice treated with vehicle **(**Fig. 7A–D**)**.

#### Topical instillation of Z-FL does not elicit gross systemic toxicity at the dose evaluated

No significant systemic changes were observed in the histological appearance of spleen, liver, and kidneys from mice treated with 200 µM of Z-FL topical eye drops relative to vehicle **(**Supplementary Table S2**)**. However, 1 mouse treated with topical Z-FL eye drops showed cytoplasmic swelling and vacuolisation and lymphocytic infiltration in the liver, a finding typical of primary SS patients^46^.

#### Basal tear production with topical instillation of Z-FL

Mice given topical Z-FL did not show a significant change in basal tear secretion across the 6-week time period, while mice treated with vehicle showed a significant reduction in basal tear secretion across the 6-week time period **(**Supplementary Fig. S8A**)**. Topical Z-FL may attenuate the reduction of basal tear secretion associated with disease progression that develops over the 6-week treatment period in vehicle-treated NOD mice. Z-FL eye drops also did not affect the corneal staining score across the treatment time period **(**Supplementary Fig. S8B**)**.

### Systemic administration of Z-FL-hydrate given intraperitoneally (i.p.)

#### Identification of Z-FL-hydrate effective dose

**Figure 8:**
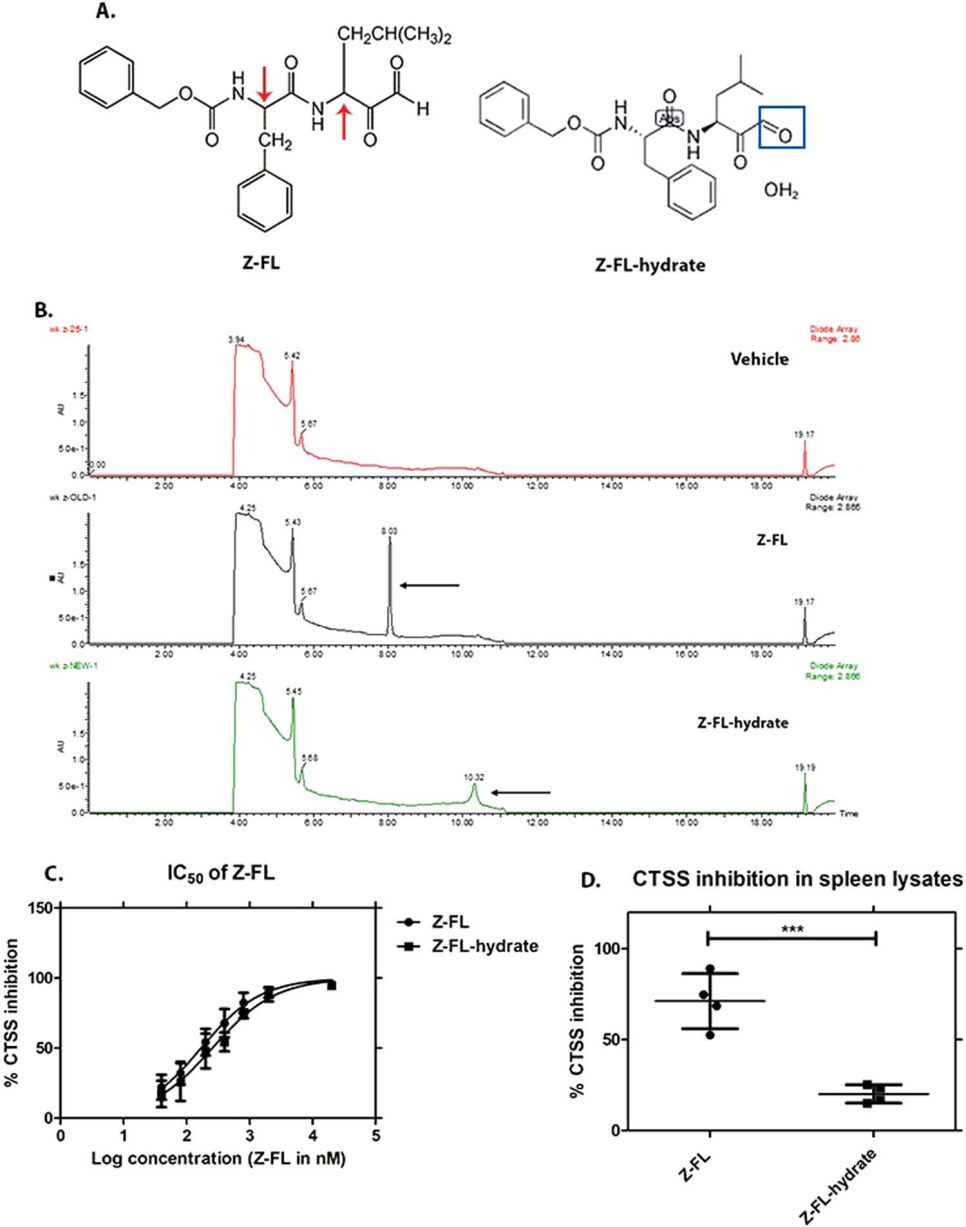
Comparison between Z-FL (Millipore) and Z-FL-hydrate (Adooq Bioscience). (**A**) Derived from a dipeptide of L-phenylalanine and L-leucine, Z-FL has two chiral centers (red arrow). In addition, Z-FL has additional modifications to the amino and carboxyl terminus. The studies in the main text were prepared using Z-FL (from Millipore, molecular weight = 424.5 g/mol); however, a second set of studies were performed using Z-FL-hydrate (from Adooq Bioscience, molecular weight = 442.5 g/mol). The increased molecular weight reflects a hydrate of the carboxyterminal aldehyde (Blue square); (**B**) To confirm relative equivalence of these two forms of Z-FL, Reverse-Phase High Performance Liquid Chromatography (RP-HPLC) was used to evaluate both in comparison to vehicle alone (25% DMSO + 75% Acetonitrile). Z-FL showed a retention time at 8.03 min, while Z-FL-hydrate showed retention time at 10.32 min. Both peaks indicated by the black arrow were collected and confirmed to have an inhibitory effect on 12.5 nM human recombinant CTSS with 96.51% and 96.26% from Z-FL and Z-FL-hydrate, respectively; (**C**) The half maximal inhibitory concentration (IC_50_) of Z-FL from both forms toward human recombinant CTSS was assessed. The IC_50_ were generally equivalent, with values of 172.5 ± 32.7 nM and 261.9 ± 59.3 nM for the batches of Z-FL and Z-FL-hydrate respectively; **(D)** The CTSS inhibitory potential of Z-FL and Z-FL-hydrate at 20 µM was also assessed in spleen lysates obtained from 16-week male NOD mice. Z-FL-hydrate showed less potent CTSS inhibition in spleen lysates compared to Z-FL (n = 4, *P* = 0.0007, data represent mean ± SD, a two-tailed, unpaired Student’s *t*-test was used to compare between groups).

Since topical Z-FL appeared to prevent the age-related loss of basal tear flow seen over 6-weeks in the NOD mouse cohort, we wanted to test whether this effect could be elicited with i.p. administration of Z-FL. We did not conduct this analysis in the initial i.p. study, and the original Z-FL used was no longer available from the vendor. As an alternative, Z-FL-hydrate was obtained from an alternative supplier (Adooq Bioscience). Through chemical analysis and evaluation of its biological potency, we determined that this compound had a ~4-fold reduced inhibitory potential for CTSS *in vitro* (Fig. 8A–D).

#### Intraperitoneal Z-FL-hydrate significantly reduces CTSS activity, lymphocytic infiltration of LG and increases basal and stimulated tear production

**Figure 9:**
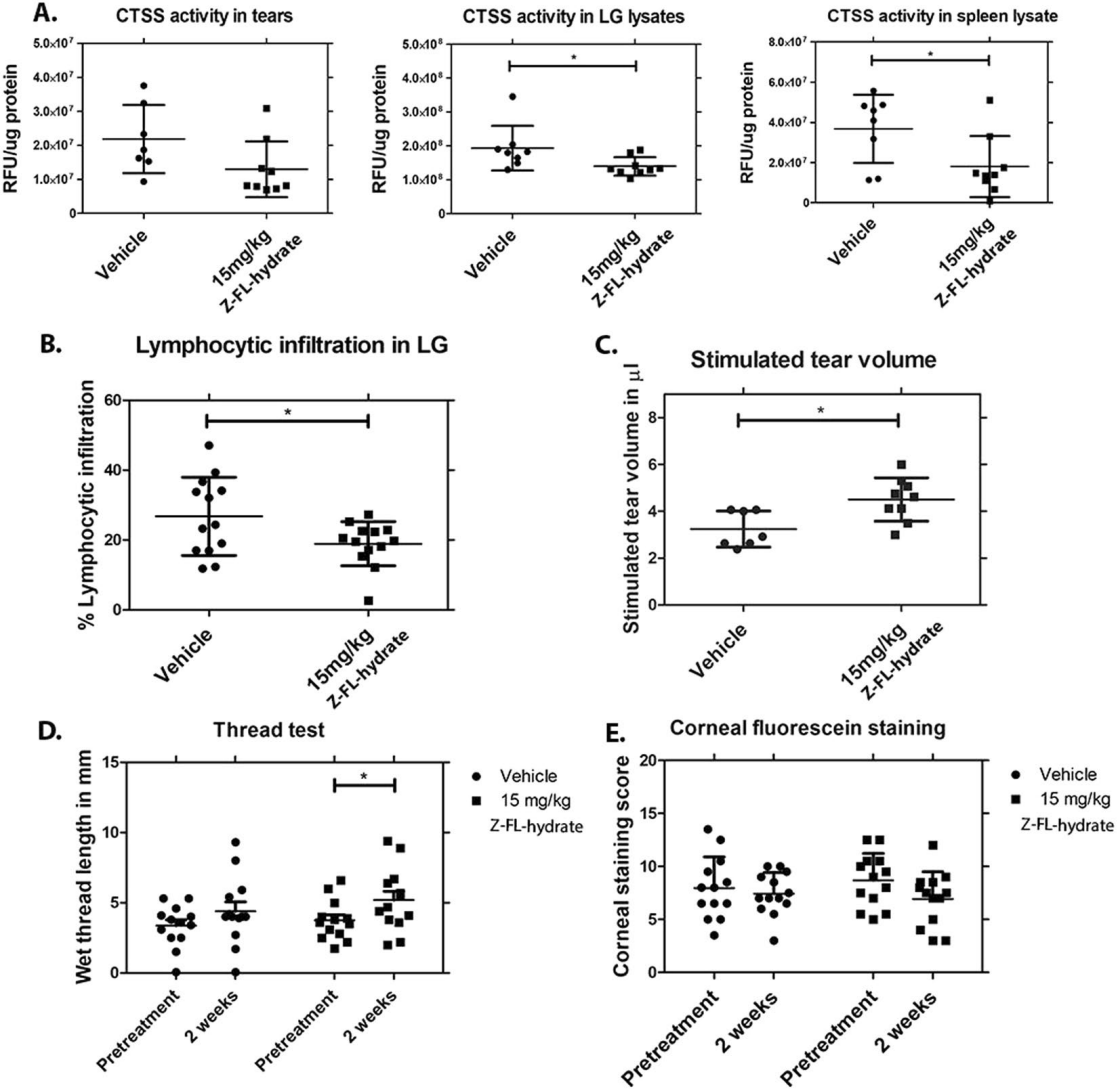
Intraperitoneal Z-FL-hydrate significantly reduces CTSS activity, lymphocytic infiltration and increases basal and stimulated tear production. 14–15 week old male NOD mice were treated every other day for 2 weeks with i.p. Z-FL-hydrate at 15 mg/kg body weight. (**A**) CTSS activity in tears (n = 7 and 9 mice in vehicle and drug groups, respectively), LG (n = 8 and 9 mice in vehicle and drug groups respectively, *P* = 0.04) and spleen lysate (n = 8 and 9 mice in vehicle and drug group, respectively, *P* = 0.03); (**B**) The percentage of LG area occupied by lymphocytic infiltration in male NOD mice treated i.p. with 15 mg/kg Z-FL-hydrate or vehicle (n = 13 mice/group, *P* = 0.04); (**C**) Stimulated tear secretion in male NOD mice treated with 15 mg/kg of Z-FL-hydrate i.p. relative to those treated with vehicle (n = 7 and 9 mice in vehicle and drug groups, respectively, *P* = 0.011); (**D**) Basal tear secretion measured by thread test at pretreatment and 2 weeks after treatment of male NOD mice with 15 mg/kg Z-FL-hydrate i.p relative to vehicle (n = 13 mice/group*, P* = 0.03 between pretreatment vs 2 weeks in drug group); (**E**) Corneal fluorescein staining at pretreatment and 2 weeks after treatment of 15 mg/kg Z-FL-hydrate i.p relative to vehicle (n = 13 mice/group). Data represent mean ± SD, a two-tailed, unpaired Student’s *t*-test was used to compare between mouse groups in (**A**–**C**), and a two-tailed, paired Student’s *t*-test was used to compare before and after treatments in each group in (**D**,**E**).

We formulated Z-FL-hydrate at 15 mg/kg, its limit of solubility in the vehicle, to obtain a roughly equipotent tissue inhibition of CTSS relative to Z-FL at 4 mg/kg. To confirm this dose was tolerated, it was administered to 14–15 week old male NOD mice every other day for 2 weeks. After 2 weeks, no notable difference in BW nor in the weight or appearance of spleen, liver or kidney in any treatment groups was observed (Supplementary Fig. S9). Also, no evidence of histological toxicity in internal organs was noted **(**Supplementary Table S3**)**. As shown in Fig. 9A, Z-FL-hydrate significantly inhibited LG and spleen CTSS, while a trend towards inhibition of tear CTSS was seen. Like Z-FL, Z-FL-hydrate significantly reduced lymphocytic infiltration of the LG (Fig. 9B) and increased stimulated and basal tear flow (Fig. 9C,D). No significant loss in basal tears was seen in the vehicle-treated group during the 2-week study; however, Z-FL-hydrate significantly improved basal tear flow during this period (Fig. 9D). On the contrary, no effect on corneal fluorescein staining was seen after 2-weeks of treatment (Fig. 9E). While validating initial findings with i.p. Z-FL in an independent cohort, this i.p. treatment also illustrates how cohort variability inherent in the strain impacts the extent of the therapeutic effect obtained^47^. For example, there was a 2-fold increase in the absolute tear CTSS activity in vehicle-treated mice from this second cohort relative to those in the Z-FL i.p. cohort in Fig. 1, and under this condition, Z-FL-hydrate given i.p. did not significantly decrease tear CTSS activity.

## Discussion

The hallmark manifestation of SS is sicca symptoms, associated with lymphocytic infiltration and reduction of acinar cells in the LG and SG^48^. Treatment options for SS are limited and focused on alleviation of symptoms, including the use of tear substitutes, short term courses of anti-inflammatory agents, or temporary occlusion of the puncta to reduce the rate of tear drainage from the eyes^1,48^. Other agents developed for treatment of non-autoimmune dry eye such as Restasis^®^ and Xiidra^®^ do not modulate mechanisms unique to autoimmune dacryoadenitis nor affect systemic disease. Consequently, identification of targets for treatment directed against specific immunopathological mechanisms of SS are of increasing importance^48^. In this study, we demonstrate that inhibition of CTSS activity with a model inhibitor, Z-FL, reduces LG inflammation and improves tear secretion in a murine model of autoimmune dacryoadenitis when disease is established, indicating the potential for CTSS inhibition as a strategy for disease treatment rather than prevention.

The longitudinal stability of the disease phenotype in the NOD mouse model of SS has previously been evaluated, with possible factors leading to variability of the SS phenotype in different mouse cohorts including genetic drift, housing, diet, water, and viral or bacterial infection^47^. In this study, the effects of systemic and topical CTSS inhibition on features of autoimmune dacryoadenitis in mice with established disease were investigated in two separate cohorts. These cohorts and the relative effectiveness of the Z-FL treatment are not directly comparable for several reasons: the ages of the mice at study termination were different (16- versus 20-weeks); the extent of LG lymphocytic infiltration can be highly variable in NOD mice^43–45^ and as shown herein by comparing vehicle-treated mice in Figs 2A and 6A for each cohort; and Z-FL bioavailability with each administration modality was not determined nor optimised. The hypothesis underlying this study is that CTSS inhibition would elicit potential therapeutic benefit in autoimmune dacryoadenitis. The two administration modalities were chosen because CTSS is elevated in autoimmune dacryoadenitis both in the LG and in the tears, and it may mediate specific pathophysiology in each domain. While systemic (i.p.) administration provides access of CTSS inhibitor to internal organs including LG, only topical administration guaranteed sustained exposure of the ocular surface and secreted tear CTSS to inhibitor.

With i.p. Z-FL, CTSS activity in tears, LG, and spleen lysates was reduced, verifying that i.p. Z-FL can inhibit CTSS activity at systemic (LG, spleen) and perhaps local levels. Inhibition of tear CTSS may be either by reduction of activity in the secreting tissue, the LG, and/or by direct access of inhibitor to tears. With i.p. administration, total lymphocytic infiltration and CD3+ cell infiltration were both significantly reduced in LG. These findings are consistent with previous reports of the inhibitory effect of RO5461111, another CTSS inhibitor, which reduced CD3+ cells in the spleen of the SLE mouse model^49^. Clik60, another CTSS inhibitor previously given i.p. in an SS mouse model, showed a decrease in autoantigen-specific T-cell proliferation in regional lymph node cells of treated mice^50^. It is possible that systemic CTSS inhibitor directly affects immune cells in the LG or alternatively affects immune cells in lymph nodes including dendritic cells which can present autoantigen to recirculating naïve T-cells, generating T-memory cells that migrate from blood into LG^51,52^. Further studies, particularly the quantification of specific T-cell markers for proliferation versus activation in LG and lymph nodes may help clarify these possible mechanisms of action of Z-FL. In addition, improved stimulated tear flow was also seen for treatment with i.p. Z-FL, an effect that may be due to the reduction of specific infiltrating immune cells into the LG, e.g., T cells. Other studies have suggested that suppression of LG lymphocytic infiltration is associated with improved tear flow^45,53–55^.

Previous studies have shown that *H2-Ab1* gene expression is upregulated in the LG of male NOD mice, in parallel with increased LG CTSS gene expression and increased LG and tear CTSS activity^39,43^. CTSS can also induce class II transactivator (CIITA), Ii, and MHC II in response to IFN-γ^39,56,57^. Reduction of *H2-Ab1* gene expression in the LG by i.p. Z-FL may reflect a specific change in a subpopulation of cells within the LG such as macrophages or DC which are particularly enriched in MHC II and reduced by Z-FL. Another CTSS inhibitor (RO5459072) reduced macrophage inflammatory responses, as demonstrated by the downregulation of MHC II and reduction of pro-inflammatory cytokines in human monocyte-derived macrophages^58^. However, systemic access of Z-FL to the draining lymph node could also account for this effect with i.p. Z-FL. Future studies on the effects of Z-FL given i.p. on immune cells in draining lymph nodes versus the LG may elucidate the mechanism of these effects.

Surprisingly, i.p. Z-FL did not affect LG gene expression of *Tnf* and *Ifng*, which are both implicated in SS pathogenesis and are highly expressed in LG and SG^39,59–61^, nor of *Ctss* and *F2rl1* gene expression, which can be induced by CTSS^20^. Previous studies have suggested that the activity and protein expression of IFN-γ and TNF-α in SG and peripheral blood are up-regulated even in the absence of lymphocytic infiltrates in SG, suggesting that these cytokines and some CTSS-related genes are not correlated directly with lymphocytic infiltration^62,63^.

In the topical Z-FL administration cohort, only tear CTSS activity was reduced, and not CTSS levels in LG and spleen. Inhibition of tear CTSS in this cohort is thus likely a local effect, occurring directly on the ocular surface, and not through inhibition of activity in the originating secreting gland, the LG. As expected, topical Z-FL did not affect LG lymphocytic infiltration, CD3+ cells in lymphocytic infiltrates, nor improve stimulated tear secretion associated with disease progression. These findings suggest the lack of effect of topical Z-FL on what may be a systemically-driven infiltration primarily sensitive to modulation of systemic (spleen, LG and/or circulating) CTSS levels with topical administration of inhibitor insufficient to achieve enough a concentration which affects target cells and organs. The ability to deliver sufficient topical Z-FL in this study was limited by Z-FL’s solubility in PBS. Improved formulation of this agent could increase the dose that may be delivered and effectively absorbed by ocular surface tissues, thus enhancing the promising effects seen with topical administration. However, topical Z-FL showed reduced *H2-Ab1* gene expression in the LG in parallel with reduced CD68+ cells in LG lymphocytic infiltrates. Since topical Z-FL had no effect on bulk lymphocytic infiltration of LG, the reduced *H2-Ab1* gene expression in topical Z-FL supports the hypothesis that reduced CD68+ cell composition of lymphocytic infiltrates may be directly responsible for reduced MHC II expression.

Topical Z-FL reduced LG gene expression of *Tnf, Ifng, Ctss*, and *F2rl1*. These findings suggest that topical Z-FL can elicit some anti-inflammatory effects in the LG that do not require systemic administration. These changes may be elicited through inhibitory effects on the production of cytokines by immune cells in draining lymph nodes that supply particular immune subpopulations to the LG. Given the effects of CTSS activity on corneal epithelial cell cytokine signaling^20^, changes in CTSS tear activity may also directly affect tear cytokines, suppressing factors that hyperactivate nerves providing a constant stimulus to the LG^64^. Overstimulation or irritation of the ocular surface can activate afferent sensory nerves that in turn activate efferent parasympathetic and sympathetic nerves supplying the LG to stimulate abnormal protein secretion^65^. Some studies have shown that increased production of pro-inflammatory cytokines in tears and LG can affect the function of both the afferent and efferent neural reflex arcs, shutting down communication between the ocular surface and the LG^64^.

As well, although inhibition of tear CTSS occurred both with topical and i.p. Z-FL, the treatment with topical Z-FL was sustained over 6-weeks while i.p. Z-FL occurred only over 2-weeks. The NOD mice in the topical cohort were older than those at the conclusion of the i.p. cohort, although not necessarily sicker. As we have noted^43,44,47^, disease development is variable across cohorts; lymphocytic infiltration and tear CTSS activity values were actually higher in these 16-week cohorts than the 20-week cohorts, although older mice may manifest other indications of disease. It is possible that the prolonged exposure of ocular surface and draining lymph nodes to CTSS inhibition is required to obtain the additional suppression of proinflammatory cytokines and other effectors of disease that did not occur with i.p. Z-FL. It is equally possible that the variability in the severity of disease development influenced this outcome. Future studies on the effects of Z-FL and other CTSS inhibitors administered topically and i.p. on cytokine expression in parallel with more extensive bioavailability measures will provide further information regarding possible differences in mechanisms of action.

Finally, i.p. Z-FL-hydrate, an alternative Z-FL chemical form, also significantly reduced CTSS activity, LG lymphocytic infiltration and increased basal and stimulated tear production. These results further support our hypothesis that inhibition of CTSS activity reduces LG inflammation and improves tear secretion in a murine model of autoimmune dacryoadenitis, indicating the potential for CTSS inhibition as a strategy for disease treatment.

CTSS inhibitors are in phase I and II clinical trials for treatment of autoimmune diseases such as rheumatoid arthritis and SS as well as other diseases associated with extracellular matrix degradation that may have autoimmune components such as abdominal aortic aneurysm. LY3000328 (www.clinicaltrials.gov, Identifier: NCT01515358) is in phase I clinical trials for abdominal aortic aneurysm treatment^66^. RWJ-445380 (www.clinicaltrials.gov, Identifier: NCT00425321) and RO5459072 (www.clinicaltrials.gov, Identifier: NCT02701985) are in phase II trials for rheumatoid arthritis and SS, respectively^67,68^. This study validates a role for CTSS activity in autoimmune dacryoadenitis in a murine model with many similarities to human SS, suggesting it as a therapeutic target for the treatment of SS.

## Materials and Methods

### Mice

Male NOD mice (NOD/MrkTac) used for all Z-FL studies were bred from Taconic breeding pairs (Hudson, NY). Male NOD mice (NOD/ShitLtJ # 001976) used for Z-FL-hydrate studies and male BALB/c mice (000651) were from The Jackson Laboratory (Sacramento, CA). Animal use followed policies approved by the University of Southern California Institutional Animal Care and Use Committee. All experiments were performed in accordance with the Guide for the Care and Use of Laboratory Animals, 8^th^ edition.

### Reagents

Z-FL-COCHO (Z-FL) and DMSO were from Millipore Sigma (Burlington, MA). Z-FL-COCHO-hydrate (Z-FL-hydrate) was from Adooq Bioscience (Irvine, CA). PEG 300 was from Spectrum (Gardena, CA) and sterile PBS was from VWR (Solon, OH). Ketamine (Ketaject^®^) was from Phoenix (St. Joseph, MO). Xylazine (AnaSed^®^) and FUL-GLO^®^ fluorescein sodium strips were from Akorn (Lake Forest, IL). Isoflurane (Fluriso^®^) was from VetOne (Boise, ID). Carbachol was from Alfa Aesar (Haverhill, MA). Free Style Lite^®^ test strips were from Abbott Diabetes Care (Alameda, CA). ZoneQuick^®^ Phenol Red threads were from Showa Yakuhin Kako Co., Ltd. (Tokyo, Japan). CytoSelect^TM^ Cell viability and Cytotoxicity assay kits were from Cell Biolabs (San Diego, CA). CTSS activity assay kits were from Biovision (Milpitas CA). Microcapillary tubes were from Drummond (Broomall, PA). The Bio-Rad protein assay kit was from Bio-Rad (Hercules, CA). KSFM, CD68 antibody (14-0681-82), AF568 goat anti-rat antibody (A11077), DAPI (62248), the Reverse Transcription Kit, TaqMan^TM^ Universal PCR Master Mix, and primers for real-time (RT)-PCR analysis including those for *Gapdh* (Mm99999915_g1), *Ctss* (Mm01255859_m1), *Tnf* (Mm00443258_m1), *Ifng* (Mm0081778_m1), *H2-Ab1* (Mm00439216_m1), and *F2rl1* (Mm00433160_m1) were from ThermoFisher Scientific (Waltham, MA). The RNeasy plus Universal Mini Kit was from Qiagen (Valencia, CA). FITC-CD3 antibody (17A2) was from Biolegend (San Diego, CA). B220 antibody (ab64100) was from Abcam (Cambridge, MA) and FITC donkey anti-rat antibody (712-095-150) was from Jackson ImmunoResearch (West Grove, PA).

### Z-FL and Z-FL-hydrate preparation

For Z-FL i.p., 4 mg/kg (2.3 mM) or 1 mg/kg (1.1 mM) of Z-FL was dissolved in 10% DMSO + 40% PEG 300 + 50% sterile PBS. For eyedrop formulation, 200 µM of Z-FL was dissolved in sterile PBS. A Branson 2510 Ultrasonic Sonicator (Danbury, CT) was used to dissolve Z-FL using 40 Hz for 2 hr at 4 °C. Formulations were prepared for use within a week and Z-FL concentrations were measured relative to a standard curve determined by RP-HPLC using a C4 column (150 × 4.6 mm, particle size 5 µm, YMC CO., LTD., Allentown, PA). The mobile phase was 10% H_2_O and 90% acetonitrile and sample was eluted at a flow rate of 1 ml/min and subjected to UV detection at 206 nm under isocratic conditions. For Z-FL-hydrate i.p., 15 mg/kg (8.6 mM) of Z-FL-hydrate was dissolved in 10% DMSO + 40% PEG300 + 50% sterile PBS and sonicated at 4 °C until the solution was clear.

### CTSS activity assays

CTSS activity with recombinant enzyme for testing Z-FL potency and in biological samples was measured using the CTSS activity assay kit following manufacturer’s instruction as described^45,69^. The fluorescence intensity of CTSS was measured using a SpectraMax iD3 (Molecular Device, San Jose, CA). CTSS activity was normalised to total protein concentration using the Bio-Rad protein assay^45^.

### *In vitro* and *in vivo* studies for determining Z-FL and Z-FL-hydrate toxicity

To estimate the MTD of Z-FL given i.p., 14-week male BALB/c mice were divided into groups and given Z-FL every other day for 2 weeks at 0.25, 0.50, 1.0, 2.0, or 4.0 mg/kg BW and compared to vehicle alone. BW of mice were monitored before each injection. After 2 weeks, BW, weights and appearances of internal organs were recorded.

*In vitro* toxicity tests for topical Z-FL used an HCE-T cell line (RIKEN BRC Cell Bank, Japan)^42^. 20, 100 and 200 µM of Z-FL were dissolved in KSFM medium plus provided supplements. HCE T-cells at 80% confluency were treated with Z-FL doses for 15 min before removal. After 8 hr, cells were again exposed to Z-FL for 15 min and then incubated in Z-FL-free KSFM medium for 16 hr, with the regimen reflecting twice-daily eyedrops. 24 hr after initial Z-FL exposure, cell viability and cytotoxicity were analysed using the CytoSelect^TM^ Cell viability and cytotoxicity assay kit with a scanning area mode of BioTek Synergy H1 Hybrid Multi-Mode Microplate Reader (Winooski, VT). KFSM- and 0.1% saponin-treated cells served as live cell and dead cell controls respectively.

Male NOD mice were used to assess the optimal dose of topical Z-FL not causing gross toxicity. Z-FL was administered topically twice-daily to 15 male NOD in 4 treatment groups of 3 mice each: 20, 100, and 200 µM Z-FL, vehicle (sterile PBS). Basal tear production and corneal fluorescein staining were measured before and after treatments.

For Z-FL-hydrate i.p., 30 male NOD mice were divided equally into 2 groups of 15 each and given either 15 mg/kg BW of Z-FL-hydrate or vehicle. Mice were injected every other day for 2 weeks. BW from each mouse was monitored every injection and after 2 weeks, weights of liver, spleen, and kidney were measured and the histotoxicological evaluation was provided as below.

### Administration of Z-FL and Z-FL-hydrate by i.p. injection in male NOD mice

30 male NOD mice were divided into 3 groups: 4 mg/kg BW, 1 mg/kg BW, and vehicle (10% DMSO + 40% PEG 300 + 50% sterile PBS). Mice were injected every other day for 2 weeks and BW monitored at each injection. Blood glucose was recorded before treatments and after the last dose in peripheral blood collected by tail nick using Free Style Lite^®^ test strips. Mice with blood glucose >250 mg/dl were considered diabetic and excluded^26^. At the end of treatments, mice were euthanised by i.p. with 50–60 mg/kg ketamine and 5–10 mg/kg xylazine, followed by cervical dislocation after stimulated tear collection. After euthanasia, one LG from each mouse was isolated for histology and fixed in 10% neutral-buffered formalin overnight at 4 °C and then, 70% ethanol. 3 randomised mice/group were selected for use of the other LG for immunofluorescence. The other LGs from the remaining mice were divided into 2 parts: ½ was used for mRNA extraction and the remaining ½ was analysed for CTSS activity. For Z-FL-hydrate i.p. studies, the same procedure was repeated as for Z-FL i.p. except 30 male NOD mice were equally divided into 2 groups and given either 15 mg/kg BW of Z-FL-hydrate or vehicle. Mice were injected every other day for 2 weeks. After 2 weeks of treatment, 2 mice/group were excluded because of diabetes. As a result, the number of mice in each group was 13 mice.

### Administration of Z-FL by topical eyedrops in male NOD mice

30 male NOD mice were given eyedrops twice daily every day for 6-weeks. Mice were divided into 2 groups (15 mice/group): 200 µM of Z-FL or vehicle (sterile PBS). Basal tear secretion, corneal fluorescein staining, and blood glucose were recorded before treatment, 3 weeks and 6 weeks. At the end of treatments, mice were euthanised and LGs were collected, divided and processed as described above for i.p.

### LG histology, immunofluorescence and quantitative analysis of inflammation

LG histology and quantification of lymphocytic infiltration was as described^43^. LG from 3 randomised mice/group were prepared for immunofluorescence staining as described^39^. FITC-CD3 antibody and B220 antibody were used at 1:50, while CD68 antibody was used at 1:100. AF568 goat anti-rat was used to detect B220, while FITC donkey anti-rat was used to detect CD68. DAPI was used to detect nuclei. Samples were imaged using a ZEISS LSM 800 confocal microscope (Carl Zeiss, Thornwood, NY).

Quantification of positively stained cells within areas of lymphocytic infiltration and the total area of the lymphocytic infiltrates used Image J software (National Institutes of Health, http://imagej.nih.gov/ij). 3 tissue sections/mouse were imaged for CD68 staining and reported as an average per mouse. 1 section/mouse was imaged for CD3 and B220 staining. In each section, 8 images were randomly acquired, and results averaged for each section. CD3+ cells and B220+ cells were calculated as the density of cells of interest in the total area of lymphocytic infiltration in each field (number of cell/mm^2^). CD68+ cells were calculated as the percentage of CD68+ labelling area normalised to total area occupied by infiltration in each field.

### Corneal fluorescein staining

Mice were anaesthetised with isoflurane and 1 µl of fluorescein sodium (FUL-GLO) was applied to both eyes^45^. A Cobalt blue light was used for illumination and the ocular surface photographed using an Excelis HD Microscope Camera with an 11.6-inch AU-600-HDS attached to a Unitron Z-8 series stereo microscope (Microscope Central, Feasterville, PA). Images were acquired from both eyes and graded by a blinded reviewer as described^45^. Corneal staining scores from both eyes are reported as an average.

### Tear collection

Basal tear secretion and stimulated tear collection were assessed as described^45^.

### Real-time PCR in LG lysates

Total mRNA was isolated by using the RNeasy Plus Universal Mini Kit. Reverse transcriptions were conducted by using TaqMan Reverse Transcription kit. Quantitative polymerase chain reaction (qPCR) was conducted using ABI 7900HT Fast Real-Time PCR System as described^45^. *Gapdh* was used as an endogenous gene and *Ctss*, *Tnf*, *Ifng*, *H2-Ab1*, and *F2rl1* were genes of interest. Reaction conditions and calculation methods were as previously^4^.

### Histotoxicological evaluation

The spleen, liver, and kidney from each mouse were collected to compare appearances and weights after treatments. Organs from 5 mice/group were randomly selected for evaluation of histological toxicity by hematoxylin-eosin (H&E) staining and all sections analysed by a blinded trained pathologist.

### Statistics

Statistical analyses were performed using GraphPad Prism (GraphPad, San Diego, CA). A two-tailed, unpaired Student’s *t*-test was used to compare between 2 independent groups. A two-tailed, paired Bonferroni-corrected Student’s *t*-test was used to compare within the same mouse before and after treatment. Differences in 3 or more independent groups were compared using one-way ANOVA with the Tukey’s multiple comparison. A repeated measures ANOVA with Tukey’s multiple comparison was used to compare between pre-, 3 week, and 6 week topical treatments within the same mouse. *P* ≤ 0.05 was considered as a significant difference.

## Supplementary information

Supplementary Figures and tables

## Data Availability

The datasets generated during the current study are available from the corresponding author on reasonable request.
