# Supplementary material for: Inhibition of Cathepsin S Reduces Lacrimal Gland Inflammation and Increases Tear Flow in a Mouse Model of Sjögren’s Syndrome
Source: Sci Rep. 2019 Jul 2;9:9559. doi: 10.1038/s41598-019-45966-7 (PMC6606642; doi:10.1038/s41598-019-45966-7)
Supplement: Supplementary file 1 — Supplementary Figures and tables [file 41598_2019_45966_MOESM1_ESM.docx]

**Inhibition of Cathepsin S Reduces Lacrimal Gland Inflammation and Increases Tear Flow in a Mouse Model of Sjögren’s Syndrome**

**Wannita Klinngam^1^, Srikanth R. Janga^2^, Changrim Lee^1^, Yaping Ju^1^, Frances Yarber^2^, Mihir Shah^2^, Hao Guo^1^, Dandan Wang^3^, J. Andrew MacKay^1,2,4^, Maria C. Edman^2^, Sarah F. Hamm-Alvarez^1, 2^***

^1^Department of Pharmacology and Pharmaceutical Sciences, School of Pharmacy, University of Southern California, Los Angeles, CA, 90033, USA

^2^Department of Ophthalmology, Roski Eye Institute, Keck School of Medicine, University of Southern California, Los Angeles, CA, 90033, USA

^3^Anatomic and Clinical Pathology, Los Angeles County + University of Southern California Medical Center, Los Angeles, CA, 90033, USA

^4^ Department of Biomedical Engineering, Viterbi School of Engineering, University of Southern California, Los Angeles, CA, 90089, USA

*Correspondence:

Sarah F. Hamm-Alvarez, Ph. D.

Department of Ophthalmology

1450 San Pablo St., #4900

Keck School of Medicine

University of Southern California

Los Angeles CA 90033

323-442-1445 Office

323-442-6412 Fax

[shalvar@usc.edu](mailto:shalvar@usc.edu)

**Supplementary Figures**


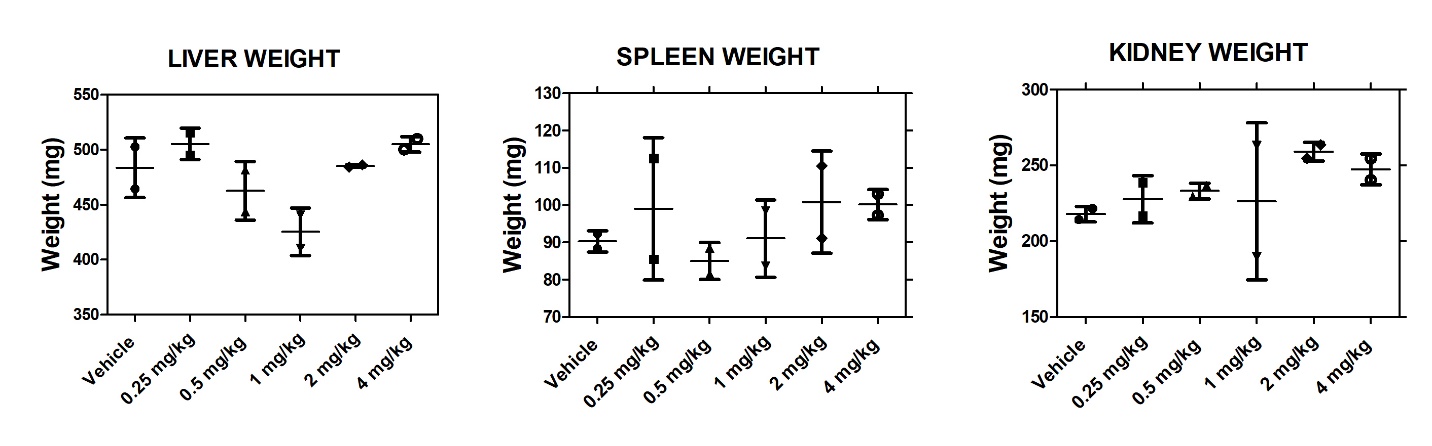


**Supplementary Figure S1. Intraperitoneal Z-FL does not elicit major changes in liver, spleen, or kidney weight.** Z-FL was injected i.p. at the indicated doses in mg/kg body weight every other day for 2 weeks starting at 14 weeks of age in BALB/c mice. (n = 2 mice/group, data represent mean ± SD).


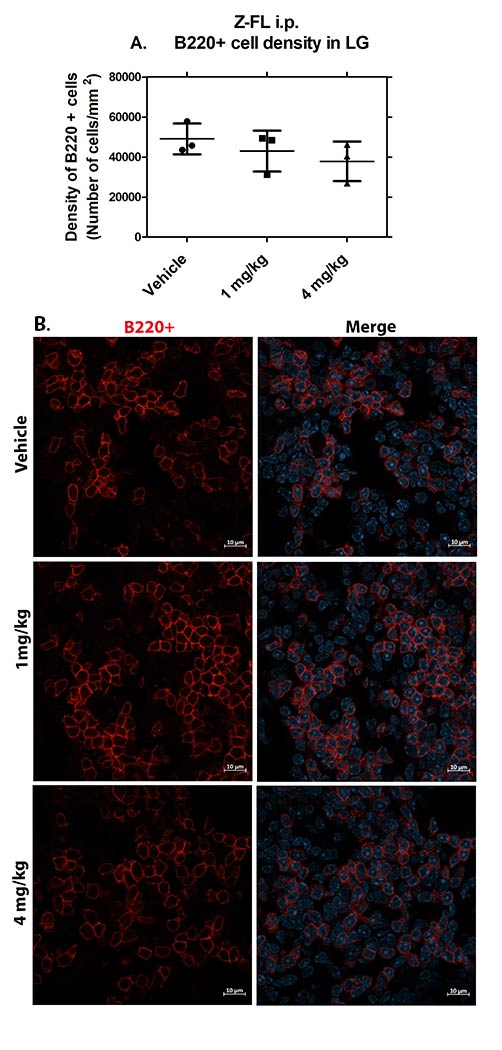


**Supplementary Figure S2. Intraperitoneal Z-FL does not alter B220+ cell abundance within the LG.** 14-15 week old male NOD mice were treated every other day for 2 weeks with i.p. Z-FL at 1, 4 mg/kg body weight. **(A)** LG were assessed for density of B220+ positive cells in areas of lymphocytic infiltration (number of cells/mm^2^). Both doses of systemic Z-FL treatment showed no difference from vehicle.; **(B)** Representative images of B220+ cell immunostaining in areas of lymphocytic infiltration from treatments in (**A**). B220 (Red) is a pan-B-cell marker, while DAPI (blue) labels nuclei. Scale bar = 10 µm. (n = 3 mice/group, data represent mean ± SD, a one-way ANOVA was used to compare between mouse groups.


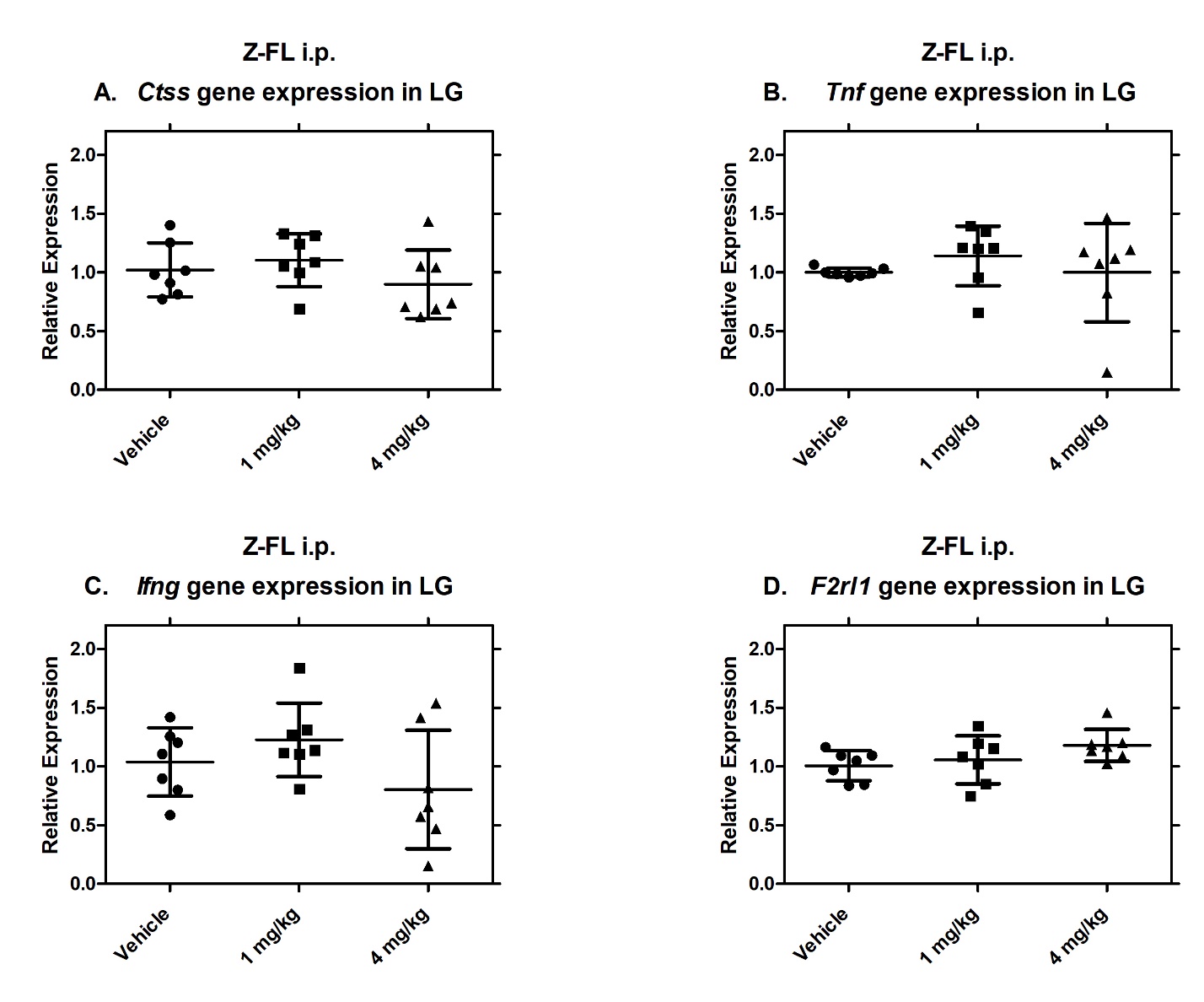


**Supplementary Figure S3. Intraperitoneal Z-FL does not affect *Ctss*, *Tnf*, *Ifng*, and *F2rl1* gene expression in LG of male NOD mice.** 14-15 week old male NOD mice were treated every other day for 2 weeks with i.p. Z-FL at 1, 4 mg/kg body weight. Gene expression levels of **(A)** *Ctss;* **(B)** *Tnf*; **(C)** *Ifng*; **(D)** *F2rl1*. Expression of genes of interest was normalised to expression of the endogenous gene, *Gapdh*. N = 7 mice/group, data represent mean ± SD, and a one-way ANOVA was used to compare between mouse groups.


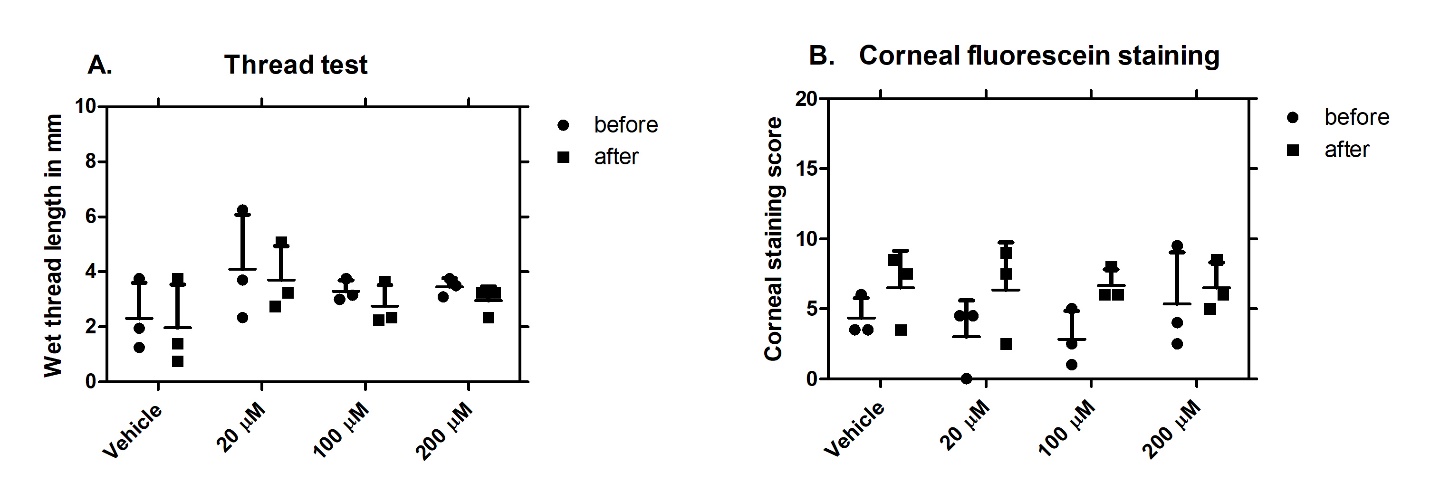


**Supplementary Figure S4. No differences in basal tear secretion or corneal surface integrity were detected after 1 week of topical Z-FL.** 14-15 week old male NOD mice were treated twice a day for 1 week with 20, 100, and 200 µM of topical Z-FL. **(A)** Basal tear secretion was measured by thread test before and after 1 week of treatment**; (B)** Corneal fluorescein staining before and after 1 week of topical Z-FL given to male NOD mice as in **(A)**. (n = 3 mice/group, data represent mean ± SD and a two-tailed, paired Bonferroni-corrected Student’s *t*-test was used to compare before and after treatments in each group).


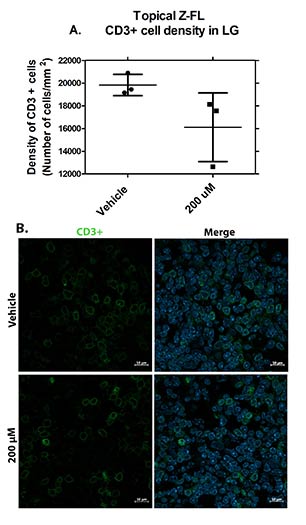


**Supplementary Figure S5. Topical instillation of Z-FL does not affect CD3+ cells within areas of lymphocytic infiltration in LG.** 14-15 week old male NOD mice were treated twice a day for 6 weeks with 200 µM Z-FL. **(A)** LG were assessed for density of CD3+ cells in areas of lymphocytic infiltration (number of cells/mm^2^). Topical treatment with Z-FL was no different from vehicle. **(B)** Representative images of CD3+ cell immunostaining in areas of lymphocytic infiltration from treatment groups in **(A)**. CD3+ (Green) is used as a total T-cell marker, while DAPI (blue) labels nuclei. N = 3 mice/group, Scale bar = 10 µm, data represent mean ± SD and a two-tailed, unpaired Student’s *t*-test was used to compare between treatment groups.


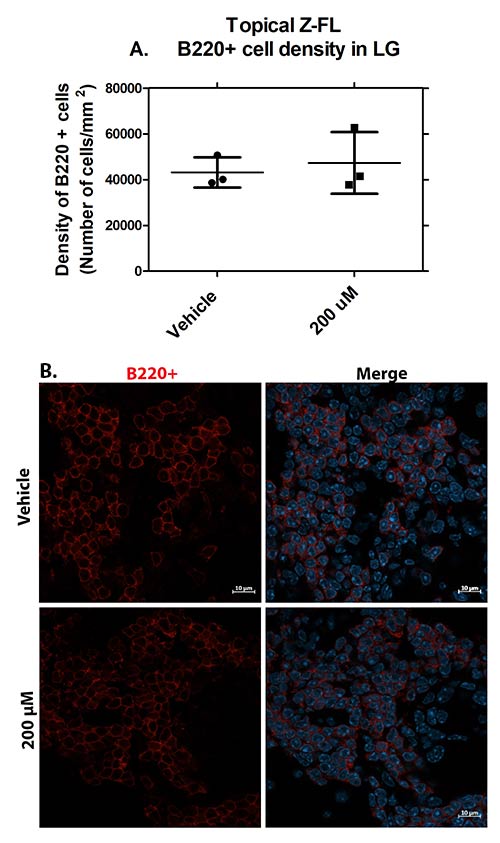


**Supplementary Figure S6. Topical instillation of Z-FL does not alter B220+ cell abundance within the LG.** 14-15 week old male NOD mice were treated twice a day for 6 weeks with 200 µM Z-FL. **(A)** LG were assessed for density of B220+ cells in areas of lymphocytic infiltration (number of cells/mm^2^). Topical treatment with Z-FL was no different from vehicle.; **(B)** Representative images of B220+ cell immunostaining in areas of lymphocytic infiltration from treatments in (**A**). B220 (Red) is used as a pan-B-cell marker, while DAPI (blue) labels nuclei. Scale bar = 10 µm. N = 3 mice/group, data represent mean ± SD, a two-tailed, unpaired Student’s *t*-test was used to compare between treatment groups.


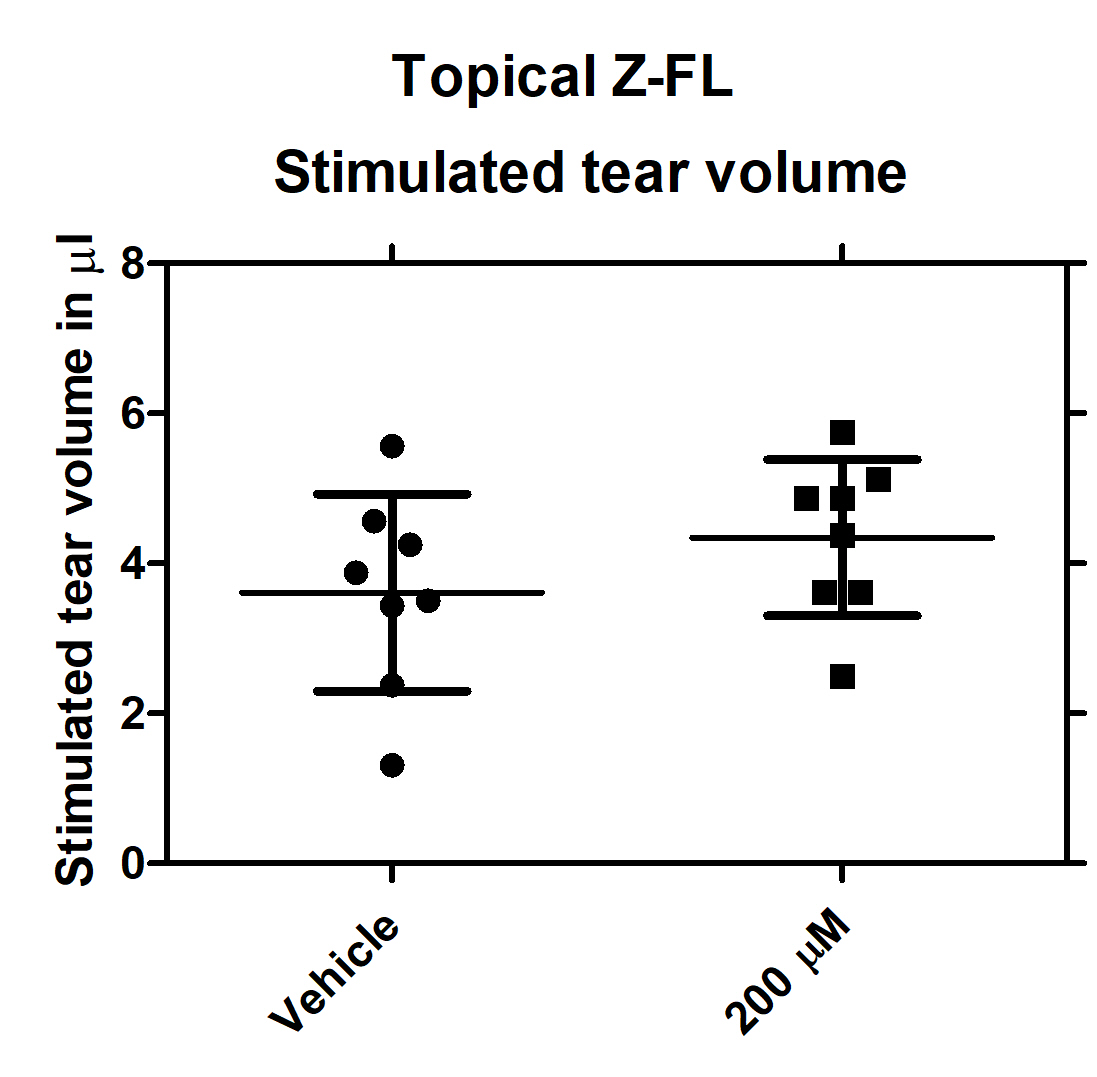


**Supplementary Figure S7. Topical instillation of Z-FL does not affect stimulated tear secretion.** 14-15 week old male NOD mice were treated twice a day for 6 weeks with 200 µM Z-FL. Carbachol-stimulated tear secretion was assessed and topical treatment with Z-FL was no different from vehicle (n = 8 mice/group). Data represent mean ± SD, a two-tailed, unpaired Student’s *t*-test was used to compare between treatment groups.


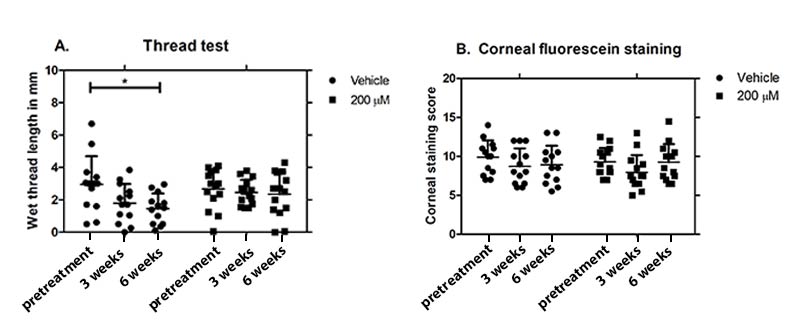


**Supplementary Figure S8. Topical instillation of Z-FL attenuates the reduction of basal tear secretion but has no measurable effect on corneal surface integrity associated with disease progression.** 14-15 week old male NOD mice were treated twice a day for 6 weeks with 200 µM Z-FL. **(A)** Basal tear secretion measured by thread test at pretreatment, 3 weeks, and 6 weeks after treatment was assessed. Vehicle showed the reduction of basal tear secretion after 6-week of treatment (*P* = 0.02), while topical treatment with Z-FL attenuated the reduction of tear secretion; **(B)** Corneal fluorescein staining at pretreatment, 3 weeks, and 6 weeks after treatment was assessed. Topical treatment with Z-FL was no different from vehicle (n = 14 mice for Z-FL and 13 mice for vehicle, data represent mean ± SD, a repeated measures ANOVA with the Tukey’s multiple comparison was used to compare within the same treatment group at different time points, and a two-tailed, unpaired Bonferroni-corrected Student’s *t*-test was used to compare 2 different mouse groups at the same time points).


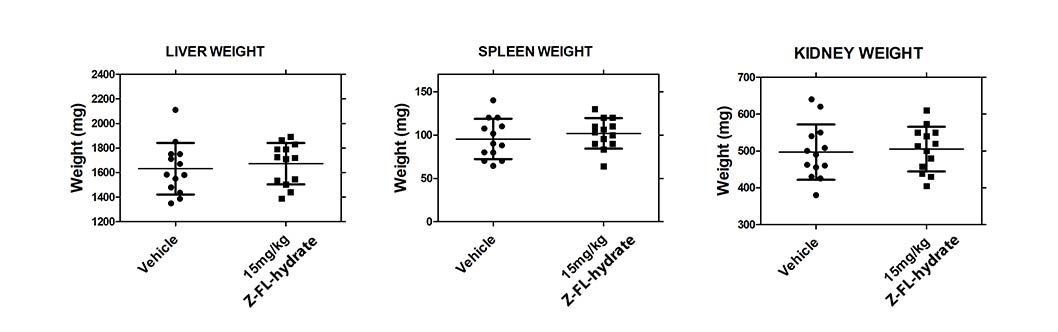


**Supplementary Figure S9. Intraperitoneal Z-FL-hydrate does not elicit major changes in liver, spleen, or kidney weight.** 14-15 week old male NOD mice were treated every other day for 2 weeks with Z-FL-hydrate i.p. at 15 mg/kg BW (n = 13 mice/group, data represent mean ± SD, and a two-tailed, unpaired Student’s *t*-test was used to compare between treatment groups).

| **Organ** | **Vehicle** | **1 mg/kg of Z-FL** | **4 mg/kg of Z-FL** |
| --- | --- | --- | --- |
| **Kidney** | H&E stained sections displayed kidney with a well-defined cortex and medulla. Numerous glomeruli were identified in the cortex. The renal medulla with loops of Henle and collecting ducts of Bellini were identified. No histopathologic abnormality was identified in the kidney. | H&E stained sections displayed kidney with a well-defined cortex and medulla. Numerous glomeruli were identified in the cortex. The renal medulla with loops of Henle and collecting ducts of Bellini were identified. One case showed mild diffuse vacuolisation of the tubular epithelial cells. No histopathologic abnormality was otherwise identified in the kidney. | H&E stained sections displayed kidney with a well-defined cortex and medulla. Numerous glomeruli were identified in the cortex. The renal medulla with loops of Henle and collecting ducts of Bellini were identified. One case showed mild diffuse vacuolization of the tubular epithelial cells.  No histopathologic abnormality was otherwise identified in the kidney. |
| **Spleen** | H&E stained sections displayed white and red pulp of the parenchyma and septa. Germinal centres and surrounding lymphocytes are identified in the white pulp area. Reticular fibres, reticulocytes, macrophages, hematopoietic cells and venous sinuses are seen in the red pulp area. These structures are essentially unremarkable. | H&E stained sections displayed white and red pulp of the parenchyma and septa. Germinal centres and surrounding lymphocytes are identified in the white pulp area. Reticular fibres, reticulocytes, macrophages, hematopoietic cells and venous sinuses are seen in the red pulp area. These structures are essentially unremarkable | H&E stained sections displayed white and red pulp of the parenchyma and septa. Germinal centres and surrounding lymphocytes are identified in the white pulp area. Reticular fibres, reticulocytes, macrophages, hematopoietic cells and venous sinuses are seen in the red pulp area. These structures are essentially unremarkable |
| **Liver** | The H&E sections displayed liver parenchyma with hepatocytes and sinusoids with Kupffer cells. Several hepatocytes were binucleated. Focal mild (1 case) cytoplasmic swelling and vacuolisation was noted. Morphology of hepatocytes is otherwise unremarkable. Portal triads with bile duct, hepatic artery and portal vein were identified and were essentially unremarkable. | The H&E sections displayed liver parenchyma with hepatocytes and sinusoids with Kupffer cells. Several hepatocytes were binucleated. Focal mild (1 case) to moderate (1 case) cytoplasmic swelling and vacuolisation was noted. Morphology of hepatocytes is otherwise unremarkable. Portal triads with bile duct, hepatic artery and portal vein were identified and were essentially unremarkable. | The H&E sections displayed liver parenchyma with hepatocytes and sinusoids with Kupffer cells. Several hepatocytes were binucleated. Focal mild (2 cases) to moderate (1 case) cytoplasmic swelling and vacuolisation was noted. Morphology of hepatocytes is otherwise unremarkable. Portal triads with bile duct, hepatic artery and portal vein were identified and were essentially unremarkable. |

**Supplementary Table S1: Histopathological report on kidney, spleen, and liver of male NOD mice after 2 weeks of Z-FL i.p. injection**

| **Organ** | **Vehicle** | **200 µM of Z-FL** |
| --- | --- | --- |
| **Kidney** | H&E stained sections displayed kidney with a well-defined cortex and medulla. Numerous glomeruli were identified in the cortex. The renal medulla with loops of Henle and collecting ducts of Bellini were identified. No histopathologic abnormality was otherwise identified in the kidney. | H&E stained sections displayed kidney with a well-defined cortex and medulla. Numerous glomeruli were identified in the cortex. The renal medulla with loops of Henle and collecting ducts of Bellini were identified. No histopathologic abnormality was otherwise identified in the kidney. |
| **Spleen** | H&E stained sections displayed white and red pulp of the parenchyma and septa. Germinal centres and surrounding lymphocytes are identified in the white pulp area. Reticular fibres, reticulocytes, macrophages, hematopoietic cells and venous sinuses are seen in the red pulp area. These structures are essentially unremarkable. | H&E stained sections displayed white and red pulp of the parenchyma and septa. Germinal centres and surrounding lymphocytes are identified in the white pulp area. Reticular fibres, reticulocytes, macrophages, hematopoietic cells and venous sinuses are seen in the red pulp area. There structures are essentially unremarkable. |
| **Liver** | The H&E sections displayed liver parenchyma with hepatocytes and sinusoids with Kupffer cells. Several hepatocytes were binucleated. Morphology of hepatocytes is generally unremarkable. Portal triads with bile duct, hepatic artery and portal vein were identified and were essentially unremarkable. | The H&E sections displayed liver parenchyma with hepatocytes and sinusoids with Kupffer cells. Several hepatocytes were binucleated. Focal mild (1 cases) cytoplasmic swelling and vacuolisation with lymphocyte infiltration was noted. Morphology of hepatocytes is otherwise unremarkable. Portal triads with bile duct, hepatic artery and portal vein were identified and were essentially unremarkable. |

**Supplementary Table S2: Histopathological report of kidney, spleen, and liver of male NOD mice after 6 weeks of topical Z-FL eyedrop treatment**

**Supplementary Table S3: Histopathological report on kidney, spleen, and liver of male NOD mice after 2 weeks of Z-FL-hydrate i.p. injection**

| **Organ** | **15 mg/kg of Z-FL-hydrate** | **Vehicle** |
| --- | --- | --- |
| **Kidney** | H&E stained sections displayed kidney with a well-defined cortex and medulla. Numerous glomeruli were identified in the cortex. The renal medulla with loops of Henle and collecting ducts of Bellini were identified. One case showed mild focal vacuolisation of the tubular epithelial cells. No histopathologic abnormality was otherwise identified in the kidney. | H&E stained sections displayed kidney with a well-defined cortex and medulla. Numerous glomeruli were identified in the cortex. The renal medulla with loops of Henle and collecting ducts of Bellini were identified. One case showed mild focal vacuolisation of the tubular epithelial cells and focal lymphocyte infiltration. No histopathologic abnormality was otherwise identified in the kidney |
| **Spleen** | H&E stained sections displayed white and red pulp of the parenchyma and septa. Germinal centres and surrounding lymphocytes are identified in the white pulp area. Reticular fibres, reticulocytes, macrophages, hematopoietic cells and venous sinuses are seen in the red pulp area. There structures are essentially unremarkable. | H&E stained sections displayed white and red pulp of the parenchyma and septa. Germinal centres and surrounding lymphocytes are identified in the white pulp area. Reticular fibres, reticulocytes, macrophages, hematopoietic cells and venous sinuses are seen in the red pulp area. There structures are essentially unremarkable. |
| **Liver** | The H&E sections displayed liver parenchyma with hepatocytes and sinusoids with Kupffer cells. Several hepatocytes were binucleated. Focal mild (2 cases) to moderate (2 case) cytoplasmic swelling and vacuolization was noted***.*** Morphology of hepatocytes is otherwise unremarkable. Portal triads with bile duct, hepatic artery and portal vein were identified and were essentially unremarkable. | The H&E sections displayed liver parenchyma with hepatocytes and sinusoids with Kupffer cells. Several hepatocytes were binucleated. Focal mild (3 cases) to moderate (2 case) cytoplasmic swelling and vacuolization was noted. Morphology of hepatocytes is otherwise unremarkable. Portal triads with bile duct, hepatic artery and portal vein were identified and were essentially unremarkable. |
